# FDMSNet: A frequency-decomposed multi-scale hybrid framework for renewable energy power forecasting

**DOI:** 10.1371/journal.pone.0356755

**Published:** 2026-10-13

**Authors:** Miao Xiao, Hongli Jiang, Nan He, Xiaobing Zhang

**Affiliations:** School of Intelligent Manufacturing, Zhejiang Dongfang Polytechnic, Wenzhou, China; PLOS: Public Library of Science, UNITED KINGDOM OF GREAT BRITAIN AND NORTHERN IRELAND

## Abstract

Accurate renewable energy power forecasting is of great significance for the safe operation, dispatch optimization, and efficient management of modern power systems. However, wind and photovoltaic power generation series usually exhibit significant non-stationarity, volatility, and multi-scale variation characteristics, posing great challenges to medium- and long-term forecasting. To address these issues, this paper proposes FDMSNet, a frequency-decomposed multi-scale hybrid network for renewable energy power forecasting. The model first divides the input sequence into non-stationary and stationary components through a frequency decomposition module. For the non-stationary component, a multilayer perceptron is adopted to extract global nonlinear variation features. For the stationary component, a multi-scale forecasting module is designed based on multi-branch downsampling and cross-scale prediction fusion. Subsequently, the forecasting results of different components are fused to generate the final power prediction. Experiments are conducted on real-world wind farm and photovoltaic power station datasets under multiple prediction horizons. MAE, MSE, and RSE are used as evaluation metrics, and FDMSNet is compared with different types of forecasting models. The experimental results show that FDMSNet achieves strong forecasting accuracy and stability in both wind and photovoltaic power forecasting tasks. Further average ranking analysis, significance testing, ablation experiments, and parameter sensitivity analysis verify the effectiveness and robustness of the frequency decomposition module, the multi-scale modeling structure, and the key parameter settings.

## Introduction

With the continuous acceleration of the transition toward low-carbon energy systems, renewable energy, especially wind and solar energy, is playing an increasingly important role in modern power systems [1]. The large-scale integration of renewable energy contributes to reducing carbon emissions, optimizing the energy structure, and promoting the green transformation of power systems. However, wind and photovoltaic power generation are highly dependent on meteorological factors such as wind speed, solar irradiance, temperature, and cloud cover, and therefore exhibit significant intermittency, volatility, and uncertainty [2,3]. These characteristics increase the complexity of power dispatch, reserve capacity allocation, and unit commitment, and may adversely affect the secure and stable operation of power systems. For example, in islanded microgrids and standalone power systems, rapid fluctuations in renewable energy output increase the regulation difficulty of power electronic devices such as inverters [4,5]. In distributed power grids, unstable power injection may cause voltage fluctuations, frequency deviations, and stability issues [6]. Meanwhile, the dynamic performance of power electronic converters can also be affected by rapid changes in renewable energy output [7]. Therefore, developing accurate, stable, and highly generalizable wind and photovoltaic power forecasting models is of great significance for improving renewable energy accommodation capacity, power system operational efficiency, and microgrid control reliability [8].

Compared with general time series, wind and photovoltaic power series usually contain long-term trends, periodic patterns, and short-term high-frequency fluctuations simultaneously. According to the forecasting time scale, power forecasting can be categorized into ultra-short-term, short-term, medium-term, and long-term forecasting, which serve different operational requirements, such as real-time regulation, power dispatching, maintenance scheduling, and power grid planning, respectively [9]. In particular, for medium- and long-term forecasting tasks, models need to accurately track the overall trend while suppressing the cumulative amplification of short-term noise over long prediction horizons. Therefore, higher requirements are imposed on trend modeling, multi-scale feature extraction, and forecasting stability [10].

In renewable energy forecasting, existing methods mainly include physical models, statistical models, and machine learning models [11]. Physical models usually describe the energy conversion process based on domain knowledge and environmental variables, such as modeling methods related to irradiance and temperature in photovoltaic performance estimation. However, these methods often rely on detailed device parameters and environmental information [12]. Statistical models represented by ARIMA and SARIMA can characterize linear correlations and partial periodic structures in historical sequences, but their adaptability to nonlinear, non-stationary, and abrupt-change patterns is limited [13].

Machine learning models can be divided into traditional machine learning models and deep learning models. Representative traditional machine learning methods include LASSO regression, kNN regression, XGBoost regression, random forest regression, support vector regression [14], and differential-evolution-based extreme learning machines [15]. These methods have relatively simple structures and high training efficiency, but their forecasting performance often depends on the quality of manually constructed features, and their ability to automatically represent multi-scale fluctuations and complex non-stationary patterns in renewable energy power series is limited. In contrast, deep learning models can automatically extract temporal features and nonlinear relationships through multi-layer networks. Among them, RNN-based models, especially LSTM, have become important tools for renewable energy forecasting, and various extension strategies have been developed around the standard LSTM framework to further improve forecasting accuracy [16]. For example, CNN-LSTM structures use convolutional layers to extract local patterns, short-term fluctuations, and neighborhood correlations, and then employ LSTM layers to learn the temporal evolution of these features, making them suitable for renewable energy series with both local variations and temporal dependencies [17]. Attention-LSTM dynamically evaluates the importance of historical hidden states through an attention mechanism, enabling the model to focus more on key time segments related to power output variations, thereby improving temporal feature identification and forecasting robustness [18]. In addition, hybrid recurrent architectures that integrate different sequence learning units can enhance temporal representation learning and have shown potential in forecasting complex energy time series [19]. Related studies have shown that LSTM-based forecasting models have been widely validated in practical scenarios such as microgrid photovoltaic and wind power forecasting, demonstrating their feasibility and effectiveness in real renewable energy operation environments [20]. However, RNN-based models rely on recurrent structures to model temporal dependencies, and are prone to long-term dependency degradation and error accumulation in long-sequence forecasting, making it difficult to stably characterize complex patterns such as trends, periodicities, and high-frequency fluctuations.

Among deep learning methods, Transformer-based models have become an important category for renewable energy forecasting due to the strong capability of self-attention mechanisms in modeling global temporal dependencies [21]. Unlike recurrent architectures, Transformers can process sequence elements in parallel and directly capture interactions between distant time steps, making them particularly suitable for renewable energy series with long-range dependencies, nonlinear fluctuations, and complex temporal variations. Existing studies have successfully applied Transformer-based models to various renewable energy forecasting scenarios, including wind power [22], solar energy [23], and hydrogen-integrated renewable energy systems [24], demonstrating their strong adaptability across different energy domains. On this basis, Informer reduces the computational burden of long-sequence modeling through an efficient attention mechanism [25], while iTransformer enhances cross-feature dependency modeling by reconstructing the interaction between variable and temporal dimensions [26]. These models have promoted the development of renewable energy forecasting by improving long-range dependency modeling, feature interaction learning, and the capture of multimodal temporal dynamics. However, Transformer-based models still face several limitations. The attention mechanism is easily affected by noise, abrupt changes, and irregular high-frequency fluctuations, and these models usually have a large number of parameters and are sensitive to data scale and hyperparameter settings. In addition, although models such as Autoformer introduce trend-seasonal decomposition, the decomposed components are not always differentially modeled according to their frequency characteristics. Therefore, for renewable energy series that contain both smooth trends and multi-scale periodic fluctuations, relying solely on attention mechanisms or coarse-grained decomposition remains insufficient [27].

Compared with Transformer-based models, hybrid frameworks based on linear modeling architectures provide a simpler and more efficient solution for renewable energy forecasting. These methods usually have the advantages of simple structure, high training efficiency, and strong interpretability, and can further improve forecasting capability through sequence decomposition or the integration of lightweight nonlinear modules. Among them, DLinear and its extended models have received extensive attention in long-sequence energy demand and renewable power generation forecasting because they can explicitly model trend and seasonal components with low computational complexity [28]. Related studies have also combined linear decomposition frameworks with modules such as Kolmogorov-Arnold Networks [29], convolutional units [9], recurrent units, and multilayer perceptrons [30] to enhance nonlinear feature learning, local pattern extraction, and sequence dependency modeling capabilities. Overall, linear-based hybrid models can achieve a good balance between forecasting accuracy and computational efficiency, making them suitable for efficient training and stable deployment scenarios. However, taking DLinear as an example, its trend-seasonal decomposition usually relies on fixed-window moving averages, which are essentially a time-domain smoothing operation [31]. When wind and photovoltaic power series simultaneously contain time-varying periodicities, multi-scale fluctuations, and irregular high-frequency disturbances, fixed windows are difficult to adapt to different frequency components. Therefore, although DLinear can achieve a preliminary separation of trends and residuals, it still lacks further fine-grained modeling capability for dynamic fluctuation patterns at different time scales in renewable energy series.

In summary, wind and photovoltaic power series are strongly affected by meteorological factors and exhibit significant volatility, non-stationarity, and multi-scale characteristics, posing great challenges to medium- and long-term forecasting. Existing methods still have limitations in long-term dependency modeling, noise suppression, adaptive decomposition, and computational efficiency. Therefore, how to construct a renewable energy power forecasting model that integrates prediction accuracy, stability, computational efficiency, and multi-scale dynamic modeling capability remains an urgent problem to be addressed.

To address the above challenges, this paper proposes a multi-scale modeling framework for medium- and long-term renewable energy power forecasting. The main contributions are summarized as follows:

- A novel Frequency-Decomposed Multi-Scale Hybrid Network (FDMSNET) is proposed for renewable energy power forecasting, providing a unified framework for both short-term and long-term wind and solar power prediction. By combining frequency decomposition with component-wise forecasting, the proposed model explicitly captures heterogeneous temporal patterns in renewable energy series.
- A multi-scale forecasting module is developed for seasonal component modeling. Through average downsampling and cross-scale prediction fusion, the module is able to capture local periodic features and multi-scale fluctuation patterns, thereby improving the representation of complex seasonal dynamics.
- Extensive experiments are conducted on real-world datasets from a wind farm and a solar power station, and MAE, MSE, and RSE are adopted as evaluation metrics to assess forecasting performance. In addition, average ranking, significance testing, ablation studies, and parameter sensitivity analyses are further performed to systematically validate the effectiveness, robustness, and practical value of the proposed model.

## Methodology

The overall framework of FDMSNET is illustrated in Fig 1. First, the raw data are divided into training, validation, and test sets in chronological order. The input sequences are then normalized to reduce the interference caused by magnitude differences during model training. In the forecasting stage, the normalized time series is fed into the model and first processed by a frequency decomposition module, which separates the original sequence into a non-stationary component and a stationary component. The non-stationary component mainly characterizes the long-term evolution and global variation of the series, while the stationary component reflects relatively stable local fluctuations and periodic patterns. For the non-stationary component, an MLP structure is employed for feature extraction and mapping, enhancing the model’s ability to capture global nonlinear variations. For the stationary component, a multi-scale forecasting module (MSF) is designed to divide the stationary information into multiple scale branches. At different scales, linear layers are used to learn local fluctuation features and cross-scale variation patterns, and the outputs from all scales are fused to obtain a more comprehensive stationary representation. Finally, the modeled non-stationary and stationary components are combined to generate the forecasting output. To comprehensively evaluate the effectiveness of the proposed method, MAE, MSE, and RSE are adopted as the main evaluation metrics, and average ranking, significance testing, ablation experiments, and sensitivity analysis are further conducted to verify the predictive performance, component contributions, and parameter robustness of the model.

**Fig 1:**
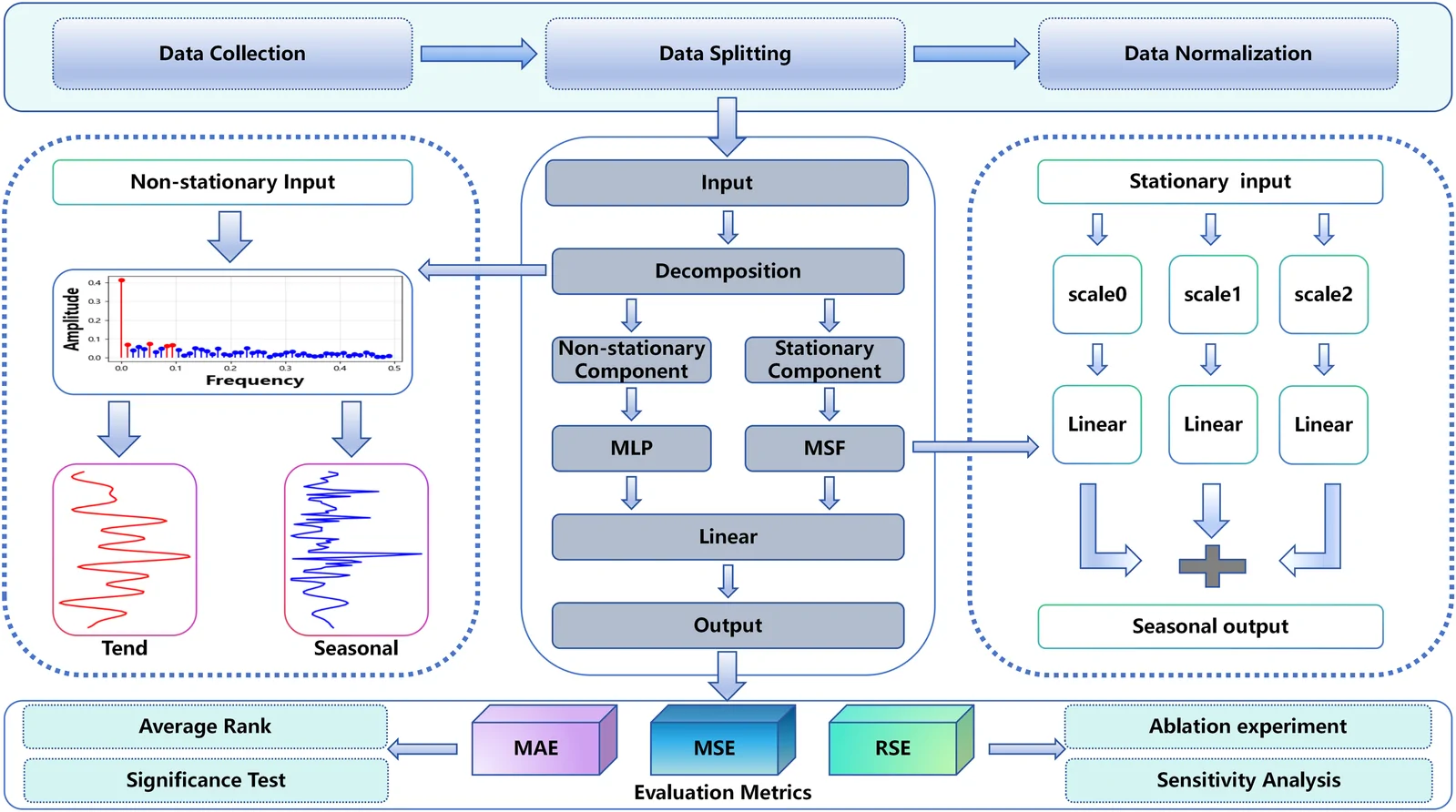
Overall workflow of FDMSNET. The collected data are chronologically split and normalized before being fed into the forecasting framework. Frequency decomposition separates the input series into a dominant non-stationary component and a stationary residual component. The non-stationary component is forecast using an MLP-based branch, whereas the stationary component is processed at multiple temporal scales by the multi-scale forecasting (MSF) module and scale-specific linear layers. The outputs of the two branches are fused through a linear layer to generate the final prediction. Model performance is evaluated using MAE, MSE, and RSE, together with average-rank analysis, significance testing, ablation experiments, and sensitivity analysis.

### Frequency decomposition

Given the input sequence $x\in {\mathbb{R}}^{B\times T\times C}$, where *B*, *T*, and *C* denote the batch size, sequence length, and number of channels, respectively, frequency decomposition is performed to separate the dominant non-stationary information from the stationary residual fluctuations.

The time-domain sequence is transformed into the frequency domain along the temporal dimension using the discrete Fourier transform (DFT):


$$\begin{array}{c} X_{f}[k]=\sum_{t=0}^{T-1}x[t]\;e^{-j2\pi kt/T},\;k=0,1,...,T-1, \end{array}$$ (1)

where $X_{f}[k]\in \mathbb{C}$ is the complex frequency coefficient. For real-valued sequences, only the first *T*/2 + 1 coefficients are retained using the real fast Fourier transform (RFFT):


$$\begin{array}{c} X_{f}=RFFT(x)\in {\mathbb{C}}^{T/2+1}. \end{array}$$ (2)

The amplitude of each frequency component is computed as


$$\begin{array}{c} A[k]=|X_{f}[k]|,\;k=0,1,...,T/2. \end{array}$$ (3)

The *k* largest amplitude components are selected as the dominant frequency set:


$$\begin{array}{c} {ℐ}_{k}=\mathrm{TopK}(A[k],k), \end{array}$$ (4)

and a binary mask is constructed to retain these components while suppressing the others:


$$\begin{array}{c} M[k]=\left\{ \begin{array}{cc} 1, & k\in {ℐ}_{k},\\ 0, & \text{otherwise}. \end{array}\right. \end{array}$$ (5)

The masked frequency representation is defined as


$$\begin{array}{c} {\tilde{X}}_{f}[k]=X_{f}[k]\cdot M[k]. \end{array}$$ (6)

The non-stationary component in the time domain is then reconstructed using the inverse RFFT:


$$\begin{array}{c} x_{non}=IRFFT({\tilde{X}}_{f})\in {\mathbb{R}}^{T}. \end{array}$$ (7)

The stationary component is obtained by subtracting the non-stationary component from the original sequence:


$$\begin{array}{c} x_{sta}=x-x_{non}. \end{array}$$ (8)

In this way, the original sequence is decomposed into a non-stationary component $x_{non}$, reconstructed from high-amplitude frequency components to capture global temporal variations, and a stationary component $x_{sta}$, containing residual fluctuations that reflect local and periodic patterns.

### Non-stationary component modeling

The non-stationary component $x_{non}$ corresponds to the dominant frequency information reconstructed from the selected high-amplitude frequency components. It mainly describes the long-term evolution and global variation of the original time series. Compared with the stationary component, the non-stationary component usually presents smoother but nonlinear temporal variations. Therefore, a lightweight MLP-based forecasting branch is adopted to map the historical non-stationary component into the prediction horizon.

As shown in Fig 2, given


$$\begin{array}{c} x_{non}\in {\mathbb{R}}^{B\times T\times C}, \end{array}$$ (9)

where *B* is the batch size, *T* is the input length, and *C* is the number of variables, the tensor is first permuted from the variable-last format to the channel-first format:


$$\begin{array}{c} z_{non}={Permute}_{\left(0,2,1\right)}\left(x_{non}\right)\in {\mathbb{R}}^{B\times C\times T}. \end{array}$$ (10)

**Fig 2:**
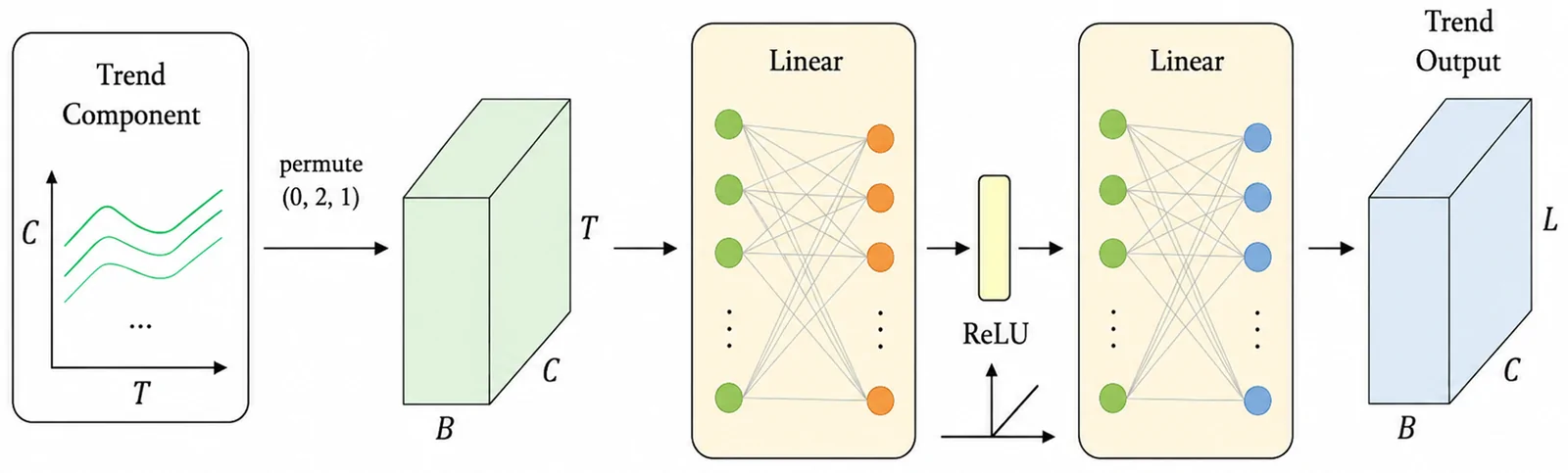
Structure of the non-stationary component forecasting branch. The non-stationary input is first permuted from B×T×C to B×C×T, allowing temporal projection to be performed independently for each variable. A two-layer MLP with a ReLU activation maps the historical sequence of length *T* to the forecasting horizon *L*, producing an output of size B×L×C. Here, *B*, *T*, *C*, and *L* denote the batch size, input length, number of variables, and prediction length, respectively.

This permutation allows the following linear layers to perform temporal projection independently for each variable.

Then, a two-layer MLP is used to transform the historical temporal length *T* into the prediction length *L*:


$$\begin{array}{c} {\hat{z}}_{non}=W_{2}\sigma \left(W_{1}z_{non}+b_{1}\right)+b_{2}, \end{array}$$ (11)

where *W*_1_ and *W*_2_ are learnable weight matrices, *b*_1_ and *b*_2_ are bias terms, and σ(·) denotes the ReLU activation function. Specifically, the two linear layers can be expressed as


$$\begin{array}{c} W_{1}\in {\mathbb{R}}^{d_{non}\times T},\;W_{2}\in {\mathbb{R}}^{L\times d_{non}}, \end{array}$$ (12)

where $d_{non}$ is the hidden dimension of the MLP and *L* is the prediction length. Accordingly, the intermediate representation and output satisfy


$$\begin{array}{c} W_{1}z_{non}+b_{1}\in {\mathbb{R}}^{B\times C\times d_{non}},\;{\hat{z}}_{non}\in {\mathbb{R}}^{B\times C\times L}. \end{array}$$ (13)

Finally, the output is permuted back to the variable-last format:


$$\begin{array}{c} {\hat{x}}_{non}={Permute}_{\left(0,2,1\right)}\left({\hat{z}}_{non}\right)\in {\mathbb{R}}^{B\times L\times C}. \end{array}$$ (14)

The non-stationary component $x_{non}$ is reconstructed from the selected high-amplitude frequency components and mainly captures the dominant global variations of the time series. To forecast this component efficiently, a lightweight MLP-based branch is used to project its historical representation into the prediction horizon.

### Stationary component modeling

After removing the non-stationary component from the original input, the residual part is regarded as the stationary component:


$$\begin{array}{c} x_{sta}=x-x_{non}, \end{array}$$ (15)

where $x_{sta}\in {\mathbb{R}}^{B\times T\times C}$, *B* is the batch size, *T* is the input length, and *C* is the number of variables. Compared with the non-stationary component, $x_{sta}$ contains relatively stable fluctuations and local periodic information. To capture stationary patterns at different temporal resolutions, a multi-scale forecasting module is designed.

The stationary component is first permuted from the variable-last format to the channel-first format:


$$\begin{array}{c} z_{sta}^{\left(0\right)}=Permute(x_{sta})={Permute}_{\left(0,2,1\right)}(x_{sta})\in {\mathbb{R}}^{B\times C\times T}. \end{array}$$ (16)

This operation changes the tensor layout from ${\mathbb{R}}^{B\times T\times C}$ to ${\mathbb{R}}^{B\times C\times T}$, so that average pooling can be conveniently performed along the temporal dimension.

The original temporal resolution is regarded as the first scale, namely Scale0. Then, average pooling with window size *w* and stride *w* is progressively applied along the temporal dimension to construct multi-scale stationary representations:


$$\begin{array}{c} z_{sta}^{\left(i\right)}={AvgPool}_{w}\left(z_{sta}^{\left(i-1\right)}\right),\;i=1,2,...,N, \end{array}$$ (17)

where *N* denotes the number of down-sampling layers. For the *i*-th scale, the temporal length is reduced to


$$\begin{array}{c} T_{i}=\frac{T}{w^{i}},\;i=0,1,...,N, \end{array}$$ (18)

with *T*_0_ = *T*. Therefore, the representation at the *i*-th scale satisfies


$$\begin{array}{c} z_{sta}^{\left(i\right)}\in {\mathbb{R}}^{B\times C\times T_{i}}. \end{array}$$ (19)

Specifically, the average pooling operation from the (i−1)-th scale to the *i*-th scale is formulated as


$$\begin{array}{c} z_{sta}^{\left(i\right)}(b,c,l)=\frac{1}{w}\sum_{m=0}^{w-1}z_{sta}^{\left(i-1\right)}(b,c,lw+m), \end{array}$$ (20)

where *b*, *c*, and *l* denote the batch index, channel index, and temporal position, respectively. This progressive down-sampling reduces the temporal resolution step by step and extracts smoother stationary patterns at coarser scales.

After obtaining the multi-scale representations, each scale is permuted back to the variable-last format:


$$\begin{array}{c} x_{sta}^{\left(i\right)}={Permute}_{\left(0,2,1\right)}\left(z_{sta}^{\left(i\right)}\right)\in {\mathbb{R}}^{B\times T_{i}\times C},\;i=0,1,...,N. \end{array}$$ (21)

Accordingly, the multi-scale stationary representations can be generally written as


$$\begin{array}{c} x_{sta}^{\left(i\right)}\in {\mathbb{R}}^{B\times \frac{T}{w^{i}}\times C},\;i=0,1,...,N. \end{array}$$ (22)

It should be noted that Fig 3 illustrates the multi-scale construction process using the case of *N* = 3 as an example, where the stationary representations are


$$\begin{array}{c} \left\{ \begin{array}{cc} x_{sta}^{\left(0\right)} & \in {\mathbb{R}}^{B\times T\times C},\\ x_{sta}^{\left(1\right)} & \in {\mathbb{R}}^{B\times \frac{T}{w}\times C},\\ x_{sta}^{\left(2\right)} & \in {\mathbb{R}}^{B\times \frac{T}{w^{2}}\times C},\\ x_{sta}^{\left(3\right)} & \in {\mathbb{R}}^{B\times \frac{T}{w^{3}}\times C}. \end{array}\right. \end{array}$$ (23)

**Fig 3:**
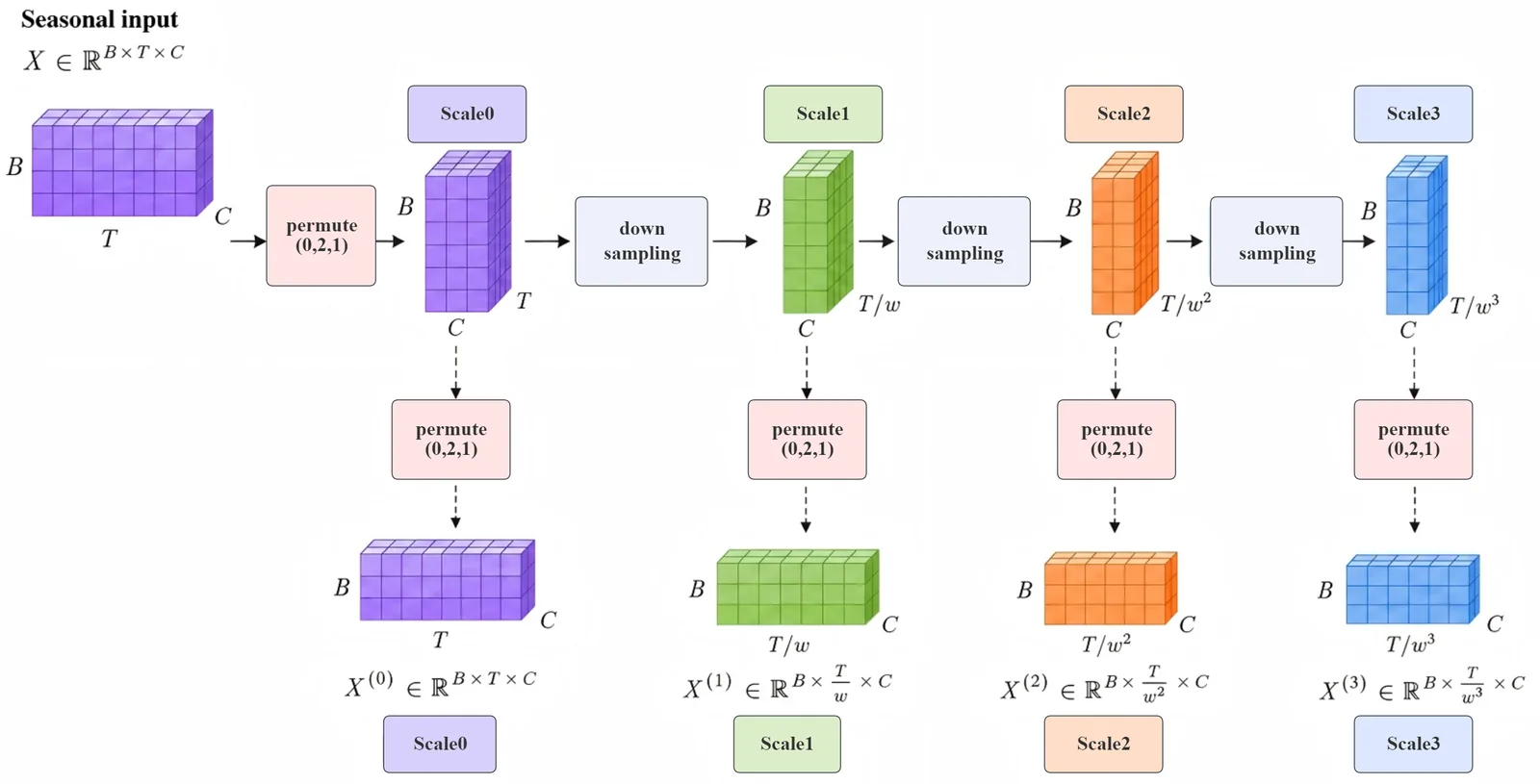
Multi-scale construction process for the stationary component. The stationary input $X\in {\mathbb{R}}^{B\times T\times C}$ is first permuted to B×C×T and then progressively downsampled along the temporal dimension using a window size and stride of *w*. Taking three downsampling layers as an example, four temporal-scale representations are constructed with lengths *T*, *T*/*w*, *T*/*w*^2^, and *T*/*w*^3^. Each representation is subsequently permuted back to the variable-last format, yielding $X^{\left(i\right)}\in {\mathbb{R}}^{B\times (T/w^{i})\times C}$ for *i* = 0,1,2,3. Here, *B*, *T*, and *C* denote the batch size, input length, and number of variables, respectively.

### Multi-scale fusion and forecasting output

For each scale, an independent linear projection layer is adopted to map the corresponding temporal representation into the prediction horizon. Let *H* denote the prediction length. The scale-specific stationary prediction is computed as


$$\begin{array}{c} {\hat{x}}_{sta}^{\left(i\right)}(b,h,c)=\sum_{l=0}^{T_{i}-1}W_{sta}^{\left(i\right)}(h,l)x_{sta}^{\left(i\right)}(b,l,c)+b_{sta}^{\left(i\right)}(h), \end{array}$$ (24)

where $W_{sta}^{\left(i\right)}\in {\mathbb{R}}^{H\times T_{i}}$ and $b_{sta}^{\left(i\right)}\in {\mathbb{R}}^{H}$ are the learnable parameters of the projection layer at the *i*-th scale. The output of each scale satisfies


$$\begin{array}{c} {\hat{x}}_{sta}^{\left(i\right)}\in {\mathbb{R}}^{B\times H\times C}. \end{array}$$ (25)

This scale-specific projection enables the model to learn both fine-grained short-term fluctuations from high-resolution representations and coarse-grained periodic patterns from low-resolution representations.

The outputs from different stationary scales are fused to obtain the final stationary prediction. In this work, an average fusion strategy is adopted:


$$\begin{array}{c} {\hat{x}}_{sta}=\frac{1}{N+1}\sum_{i=0}^{N}{\hat{x}}_{sta}^{\left(i\right)}. \end{array}$$ (26)

This fusion strategy integrates complementary information from multiple temporal resolutions and improves the robustness of stationary component forecasting.

Finally, the predicted non-stationary component and stationary component are added to generate the final forecasting result:


$$\begin{array}{c} \hat{y}={\hat{x}}_{non}+{\hat{x}}_{sta}. \end{array}$$ (27)

By separately modeling the non-stationary and stationary components, FDMSNET effectively captures both global temporal evolution and local periodic fluctuations, thereby improving the overall forecasting performance.

## Experiment

### Data description and preprocessing

#### Data description.

This study employs two publicly available datasets released by the State Grid Corporation of China, including one solar power dataset and one wind power dataset [32].The solar dataset is collected from Solar Station Site 3 with a nominal capacity of 30 MW, while the wind dataset is collected from Wind Farm Site 1 with a nominal capacity of 99 MW. Both datasets are recorded at 15-minute intervals and contain synchronized meteorological observations and renewable energy generation measurements. The solar dataset covers the period from January 1, 2019 to July 31, 2019, with 20,352 samples. It includes timestamp, total solar irradiance (TSI), direct normal irradiance (DNI), global horizontal irradiance (GHI), atmospheric pressure (Air_P), relative humidity (Air_H), and power output. The wind dataset covers the period from January 1, 2019 to December 31, 2020, with 70,176 samples. It includes timestamp, wind speed and wind direction at 10 m, 30 m, 50 m, and hub height, air temperature (Air_T), atmospheric pressure (Air_P), relative humidity (Air_H), and power output. For the wind variables, WS_*x* and WD_*x* denote wind speed and wind direction measured at *x* meters above the ground, respectively.The datasets are publicly available at https://github.com/Bob05757/Renewable-energy-generation-input-feature-variables-analysis/tree/main.

Table 1 summarizes the data scale and temporal coverage of the two datasets. The solar power output has a mean value of 5.449 MW and a standard deviation of 8.259 MW, with a maximum value of 29.911 MW. The wind power output has a mean value of 23.427 MW and a standard deviation of 24.130 MW, with a maximum value of 98.094 MW.

**Table 1:** Summary of the renewable energy datasets used in this study.

Dataset	Capacity (MW)	Samples	Variables	Period	Resolution
Solar station site 3	30	20,352	6	2019-01-01 to 2019-07-31	15 min
Wind farm site 1	99	70,176	12	2019-01-01 to 2020-12-31	15 min

As shown in Fig 4, the two datasets exhibit distinct temporal characteristics: solar power is strongly affected by irradiance variation and daytime availability, whereas wind power shows more irregular fluctuations driven by changes in wind speed.

**Fig 4:**
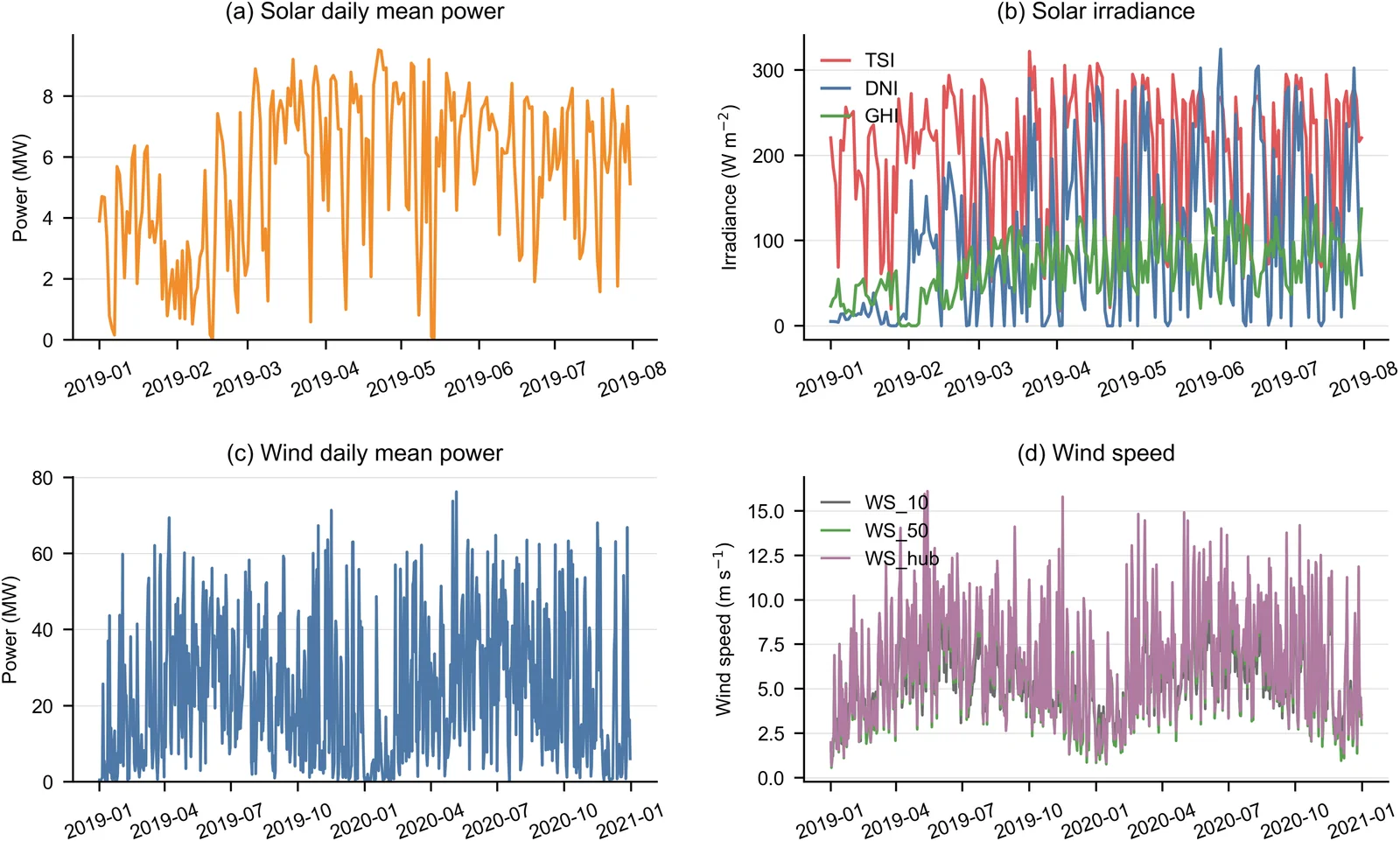
Temporal patterns of the solar and wind power datasets. Daily mean power output and the principal meteorological drivers are shown over time for the photovoltaic station and wind farm.

Fig 5 further shows that both power series are right-skewed, with many low-output observations, which increases the difficulty of accurate renewable energy forecasting.

**Fig 5:**
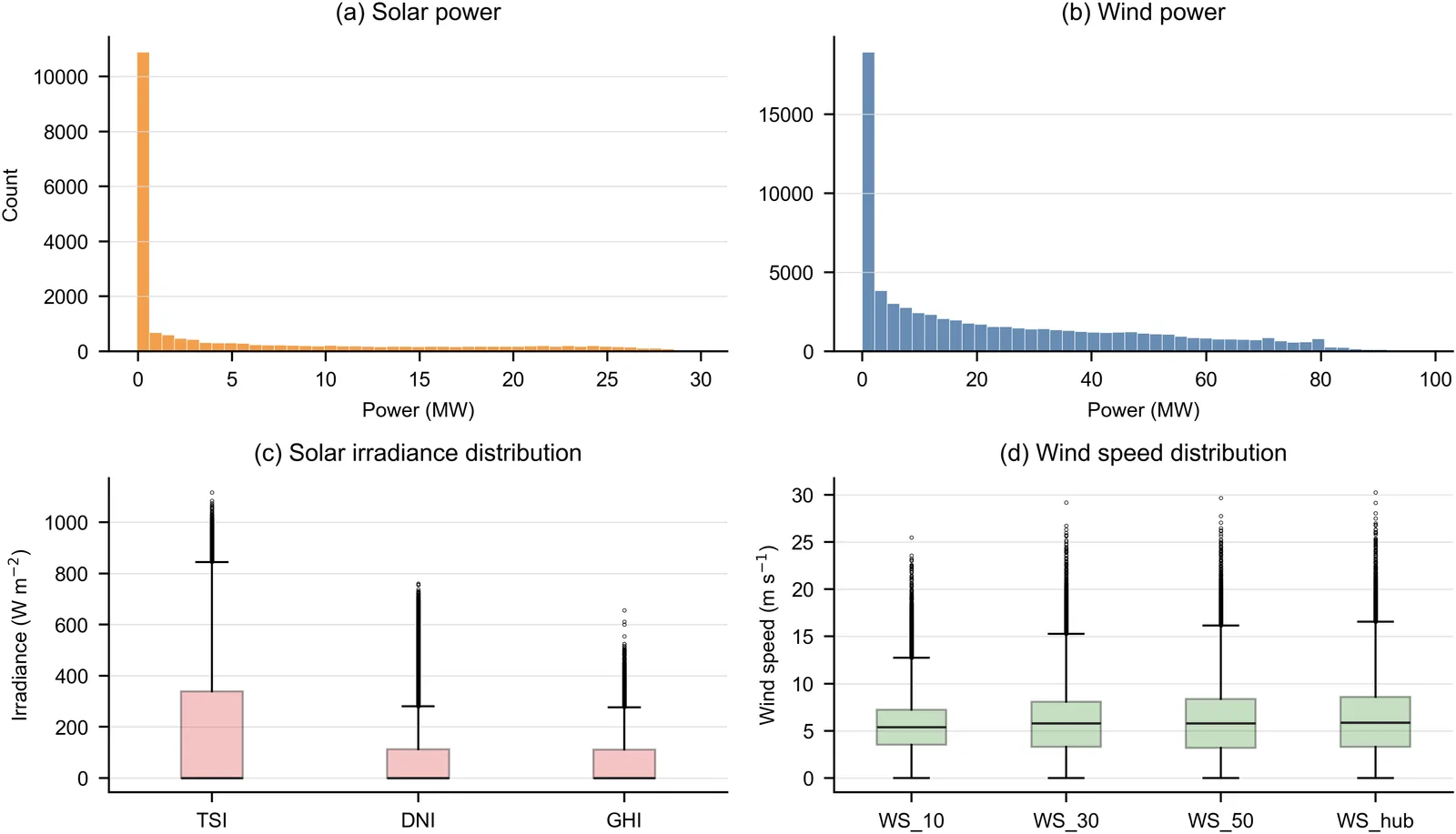
Distributions of power output and key meteorological variables. Histograms summarize the empirical distributions of power output and the main irradiance- and wind-related predictors in the two datasets.

The correlation analysis in Fig 6 indicates that solar power is positively correlated with TSI, DNI, and GHI, with correlation coefficients of 0.71, 0.54, and 0.68, respectively. Wind power is strongly correlated with wind speed at different heights, with coefficients ranging from 0.80 to 0.86. These statistical properties demonstrate that the datasets contain nonlinear, intermittent, and meteorology-dependent patterns, making them suitable for evaluating the proposed model under different renewable energy forecasting scenarios.

**Fig 6:**
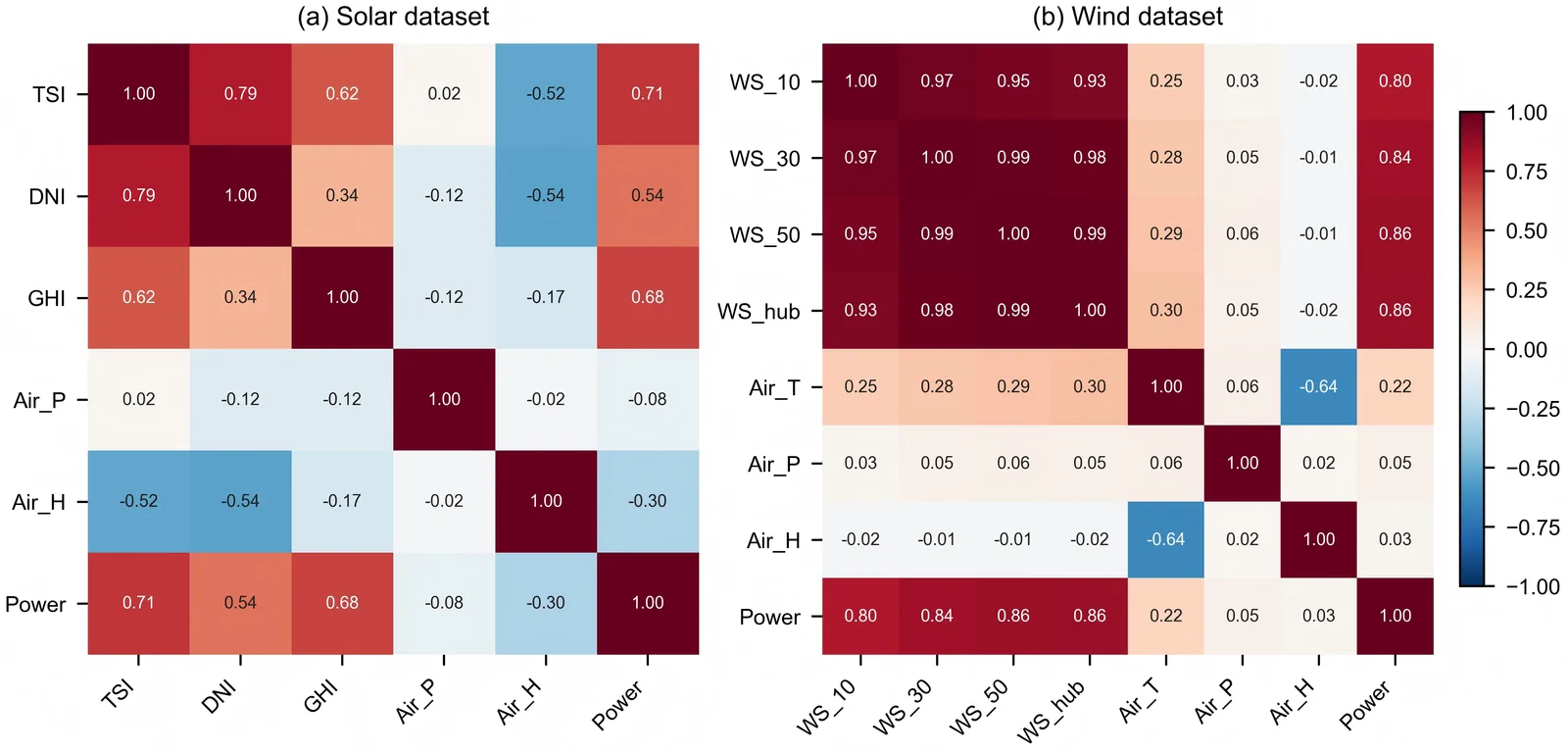
Correlations between meteorological variables and power output. Pearson correlation matrices are shown separately for the solar and wind datasets; color intensity represents the magnitude and sign of each pairwise correlation coefficient.

### Data preprocessing

The dataset is first sorted in chronological order and then divided into the training, validation, and test sets with a ratio of 7:1:2. To reduce the influence of scale differences among variables and improve training stability, all features are standardized before being fed into the model. Specifically, for each feature dimension, the mean and standard deviation are first computed from the training set as


$$\begin{array}{c} \mu_{j}=\frac{1}{N_{train}}\sum_{i=1}^{N_{train}}x_{i,j}, \end{array}$$ (28)

$$\begin{array}{c} \sigma_{j}=\sqrt{\frac{1}{N_{train}}\sum_{i=1}^{N_{train}}{\left(x_{i,j}-\mu_{j}\right)}^{2}+\epsilon }, \end{array}$$ (29)

where $x_{i,j}$ denotes the value of the *j*-th feature of the *i*-th sample in the training set, $N_{train}$ is the number of training samples, and ϵ is a small constant to ensure numerical stability. Then, the training, validation, and test sets are normalized using the statistics of the training set:


$$\begin{array}{c} {\tilde{x}}_{i,j}=\frac{x_{i,j}-\mu_{j}}{\sigma_{j}}. \end{array}$$ (30)

In this way, the validation set and test set share the same normalization parameters as the training set, which avoids information leakage and ensures a fair evaluation of model performance.

### Evaluation metrics

To quantitatively assess the forecasting performance of the proposed model and the baselines, three widely used error metrics are adopted: Mean Absolute Error (MAE), Mean Squared Error (MSE), and Root Squared Error (RSE).

Given the predicted values $\hat{y}$ and the ground-truth values *y*, these metrics are defined as follows:

- **Mean Absolute Error (MAE)** calculates the average absolute difference between predictions and ground-truth:

$$\begin{array}{c} MAE=\frac{1}{n}\sum_{i=1}^{n}|{\hat{y}}_{i}-y_{i}|. \end{array}$$ (31)

- **Mean Squared Error (MSE)** calculates the average squared difference between predictions and ground-truth:

$$\begin{array}{c} MSE=\frac{1}{n}\sum_{i=1}^{n}({\hat{y}}_{i}-y_{i}{)}^{2}. \end{array}$$ (32)

- **Root Squared Error (RSE)** measures the ratio of the prediction error to the deviation of the ground-truth from its mean:

$$\begin{array}{c} RSE=\frac{\sqrt{\sum_{i=1}^{n}({\hat{y}}_{i}-y_{i}{)}^{2}}}{\sqrt{\sum_{i=1}^{n}(y_{i}-\bar{y}{)}^{2}}}, \end{array}$$ (33)

where $\bar{y}=\frac{1}{n}\sum_{i=1}^{n}y_{i}$ is the mean of the ground-truth values.

These metrics jointly evaluate the model’s accuracy, stability, and the relative error magnitude, providing a comprehensive assessment of forecasting performance across different horizons and datasets. All error metrics reported in this study are calculated and presented on the standardized scale.

### Experimental settings

#### Baseline models.

To comprehensively evaluate the performance of **FDMSNET**, we select a set of representative forecasting methods as comparative baselines. These baselines encompass recurrent neural networks (RNNs), linear models, Transformer-based architectures, and Lastest time-series forecasting models.

The selected comparative models are listed as follows:

- **LSTM** [33]: a classical recurrent neural network capable of capturing long-term temporal dependencies in time-series data.
- **Bi-LSTM** [34]: a bidirectional variant of LSTM that leverages both past and future information for improved sequence modeling.
- **DLinear** [31]: a linear model designed for efficient time-series forecasting with decomposition-based feature extraction.
- **Autoformer** [35]: a Transformer variant with a decomposition architecture and Auto-Correlation mechanism, enabling progressive decomposition of complex time series and efficient sub-series dependency modeling.
- **Informer** [36]: an efficient Transformer for long-sequence forecasting, featuring ProbSparse self-attention (O(LlogL)), attention distilling to reduce layer inputs, and a generative decoder that predicts entire sequences in one forward pass, improving inference speed and accuracy.
- **iTransformer** [37]: a Transformer variant applying attention and feed-forward networks on inverted dimensions, embedding time points as variate tokens to capture multivariate correlations and learn nonlinear representations, achieving state-of-the-art performance on real-world datasets.
- **TimeFilter** [38]: a GNN-based framework for adaptive and fine-grained dependency modeling in time series. It constructs a graph from input sequences and filters out irrelevant correlations while preserving critical patch-specific dependencies, addressing limitations of coarse-grained channel clustering and improving generalization..
- **WPMixer** [39]: an MLP-based model for long-term time series forecasting that integrates multi-resolution wavelet decomposition, patching with embedding, and MLP mixing. Wavelet decomposition extracts frequency and time-domain features, patching captures extended local context, and MLP mixing incorporates global information.

By covering a diverse range of modeling paradigms, these baselines enable a comprehensive comparative analysis of the proposed method in terms of predictive accuracy, the ability to capture temporal dependencies, and multi-scale feature extraction capabilities.A summary of the model categories and their key hyperparameters is provided in Table 2.

**Table 2:** Model categories, models, and parameters.

Category	Model(year)	Parameters
RNN	LSTM (1997)	hidden_size=200, num_layers=2, batch_first=True, learning_rate=0.001
	BiLSTM (1997)	hidden_size=200, num_layers=2, batch_first = True, learning_rate=0.001
Linear	DLinear (2023)	moving_avg=25, individual=False, learning_rate = 0.001
Former	Transformer (2017)	d_model = 512, d_ff = 2048, n_heads = 8, e_layers = 2, d_layers = 1, learning_rate = 0.001
	Autoformer (2021)	moving_avg = 25, d_model = 512, d_ff = 2048, n_heads = 8, e_layers = 2, d_layers = 1, learning_rate = 0.001
	Informer (2021)	d_model = 512, d_ff = 2048, n_heads = 8, e_layers = 2, d_layers = 1, learning_rate = 0.001
	iTransformer (2023)	d_model = 512, d_ff = 2048, n_heads = 8, e_layers = 2, d_layers = 1, learning_rate = 0.001
Latest	TimeFilter (2025)	d_model=512, d_ff=2048, patch_len=16, learning_rate=0.001
	WPMixer (2025)	tfactor = 5, dfactor = 5, wavelet = ’db2’, level = 1, stride = 8, no_decomposition = False, learning_rate = 0.001
Ours	FDMSNET(2026)	top_k = 8 for wind, top_k = 1 for solar, down_sampling_layers = 3, down_sampling_window = 2, learning_rate = 0.001

#### Experimental environment.

All experiments were conducted on a workstation with the following hardware configuration. The system is powered by an AMD Ryzen 7 7800X3D 8-core processor and equipped with an NVIDIA GeForce RTX 4090 GPU and CUDA 12.2 support.The system memory consists of four DDR5 DIMM modules, each with a capacity of 32 GB, providing a total of 64 GB of RAM.The software environment includes Python 3.9.21 and PyTorch 2.7.0 + cu126.

This experimental setup provides sufficient computational resources for training and evaluating large-scale deep learning models for time-series forecasting tasks, ensuring reproducibility and high-performance execution of the proposed framework.

## Discussion

### Sensitivity analysis

To further verify the influence of the Top_k parameter in the frequency decomposition module on the forecasting performance of FDMSNET, this study conducts a hyperparameter sensitivity analysis on both the wind and solar datasets. This experiment aims to investigate the performance variation of the model under different Top_k values across multiple prediction horizons, identify the reasonable value range and stable interval of Top_k under different renewable energy scenarios, and provide experimental evidence for parameter settings in subsequent comparative experiments, thereby ensuring the accuracy, stability, and comparability of the model in wind and solar power forecasting tasks.

In the sensitivity analysis, only the Top_k parameter in the frequency decomposition module is varied, while the remaining model structure and core training parameters are kept unchanged. The value of Top_k is set from 0 to 32, and experiments are conducted on the wind farm dataset and the solar power station dataset, respectively. Model performance is evaluated using three metrics: MAE, MSE, and RSE, under four prediction horizons of 96, 192, 336, and 720.

As shown in Fig 7, on the wind dataset, when Top_k gradually increases from 0, MAE, MSE, and RSE all show a clear downward trend, indicating that introducing frequency components can effectively improve the model’s ability to capture wind power fluctuation patterns. As Top_k continues to increase, the model performance gradually becomes stable. Across different prediction horizons and evaluation metrics, better results are mainly concentrated in the range of Top_k from 8 to 13. When Top_k is greater than 8, the variation in each metric becomes relatively small, indicating that the model has entered a stable performance interval.

**Fig 7:**
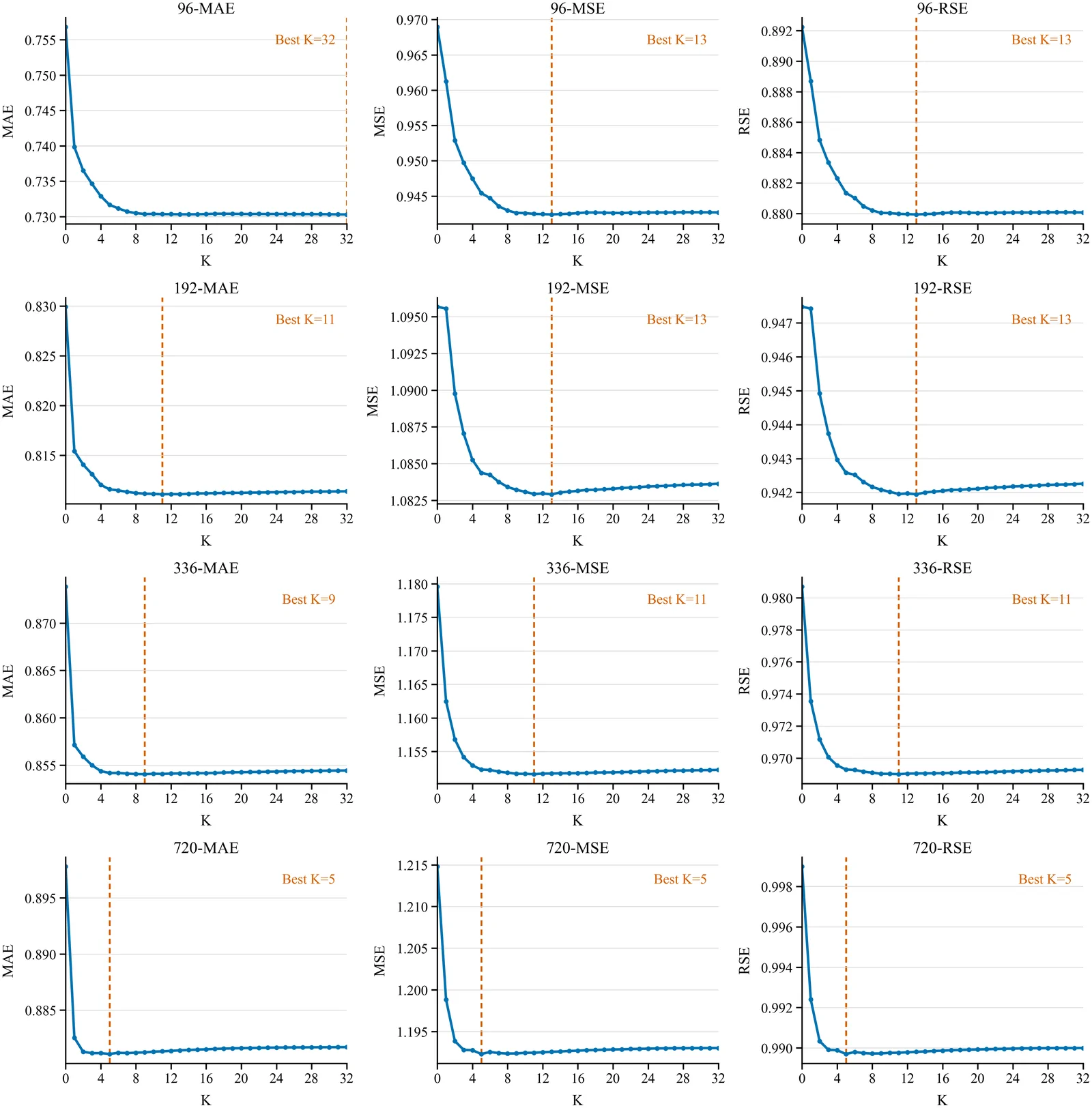
Sensitivity of wind-power forecasting to the Top_k parameter. MAE, MSE, and RSE are plotted against Top_k for prediction horizons of 96, 192, 336, and 720; lower values indicate better forecasting performance.

As shown **in** Fig 8, the trend on the solar dataset is more concentrated. When Top_k increases from 0 to 1, the model performance improves most significantly, with MAE, MSE, and RSE all decreasing substantially. Under the four prediction horizons, Top_k = 1 generally achieves the best results. Further increasing Top_k does not lead to obvious additional improvement, and some metrics even show slight fluctuations. Therefore, for the solar dataset, Top_k = 1 is the optimal choice, while Top_k values from 1 to 10 remain generally stable.

**Fig 8:**
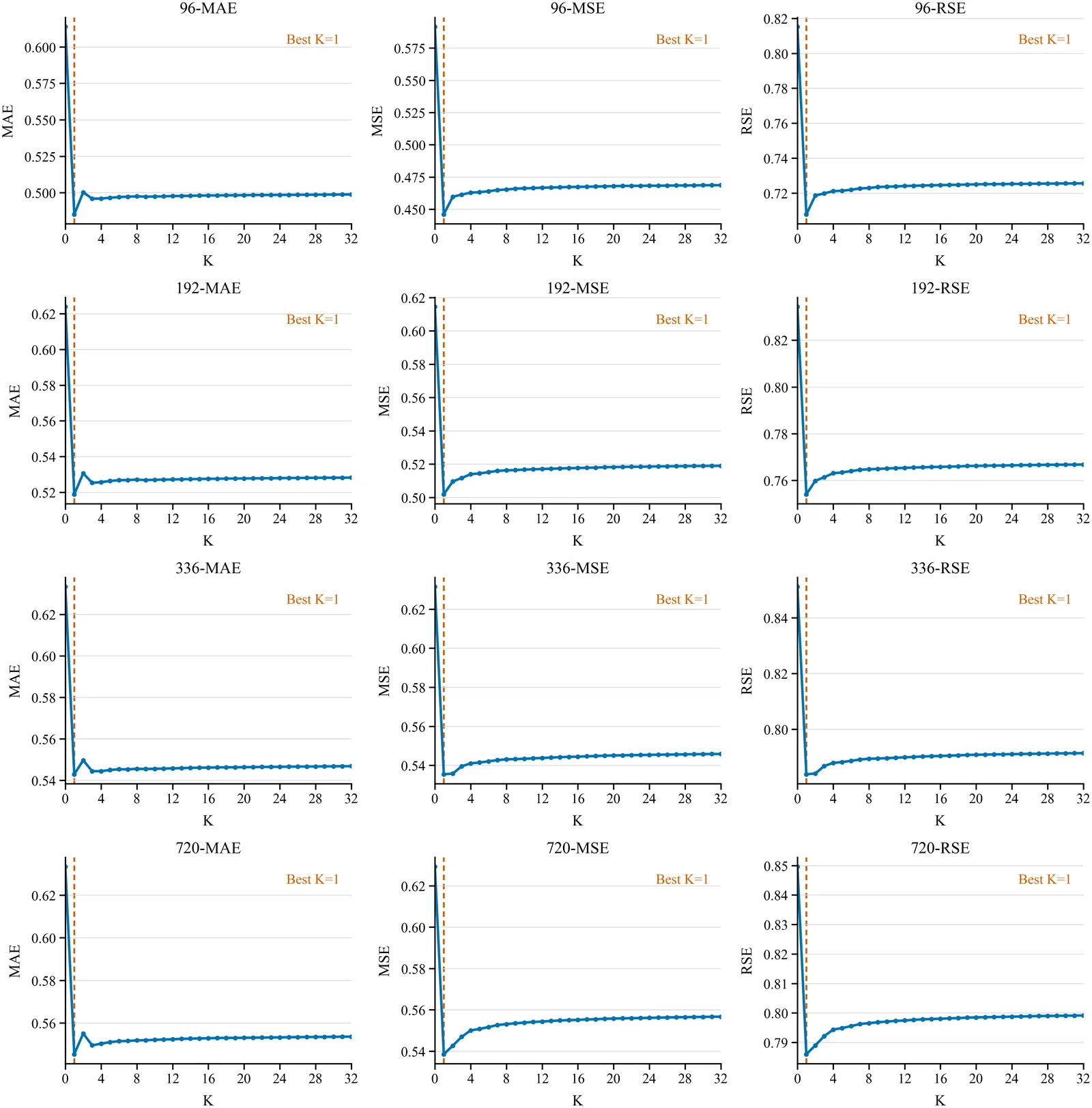
Sensitivity of solar-power forecasting to the Top_k parameter. MAE, MSE, and RSE are plotted against Top_k for prediction horizons of 96, 192, 336, and 720; lower values indicate better forecasting performance.

In summary, the Top_k parameter has an important impact on the forecasting performance of FDMSNET, but its optimal value is closely related to the data type. For the wind dataset, the model performs better when Top_k is between 8 and 13, and enters a stable interval when Top_k is greater than 8. Therefore, Top_k can be set to 8 in subsequent wind power comparative experiments to balance forecasting accuracy and computational efficiency. For the solar dataset, Top_k = 1 achieves optimal and stable forecasting performance; therefore, Top_k = 1 is adopted as the standard configuration in subsequent solar power comparative experiments.

### Comparative experiments

To comprehensively evaluate the effectiveness, stability, and generalization ability of FDMSNET in renewable energy power forecasting tasks, comparative experiments are conducted on both the wind and solar datasets. The purpose of this experiment is to systematically compare FDMSNET with different types of mainstream time series forecasting models, thereby verifying the overall forecasting advantages of the proposed model from the perspectives of multiple prediction horizons and multiple evaluation metrics. Meanwhile, to reduce the randomness caused by a single initialization and provide sufficient and reliable experimental results for subsequent average ranking and significance analyses, experiments are repeated with multiple random seeds to enhance the stability and statistical credibility of the conclusions. The comparative experiments are performed on the wind farm dataset and the solar power station dataset, with prediction horizons set to 96, 192, 336, and 720. The models involved in the comparison and their core parameter settings are consistent with Table 2. MAE, MSE, and RSE are adopted as evaluation metrics, where lower values indicate better forecasting performance.

### Wind power forecasting

As shown in Table 3, FDMSNET demonstrates strong overall forecasting capability on the wind power dataset across three random seeds and four prediction horizons. To provide a more comprehensive evaluation, the model performance is first compared from the perspective of the average errors of different model categories. The results show that, compared with recurrent neural network models, FDMSNET reduces the average MAE, MSE, and RSE by 9.59%, 6.09%, and 3.17%, respectively. Compared with the linear model, the average MAE, MSE, and RSE are reduced by 0.68%, 0.95%, and 0.48%, respectively. Compared with Transformer-based models, the corresponding reductions are 8.06%, 20.63%, and 10.66%. Compared with recent advanced time series forecasting models, the average MAE, MSE, and RSE are reduced by 3.84%, 17.29%, and 8.90%, respectively. These results indicate that FDMSNET not only achieves clear advantages over complex Transformer-based models, but also remains highly competitive against recurrent neural network models, the linear model, and recent advanced forecasting models.

**Table 3:** Performance comparison of different models on the wind power dataset across three random seeds.

Seed	Model	MAE				MSE				RSE			
		96	192	336	720	96	192	336	720	96	192	336	720
42	LSTM	0.842	0.879	0.899	0.920	1.008	1.105	1.147	1.182	0.910	0.952	0.967	**0.985**
	BiLSTM	0.843	0.890	0.909	0.922	1.005	1.135	1.157	1.185	0.909	0.964	0.971	0.987
	DLinear	0.712	0.801	0.852	0.886	0.904	1.078	1.140	1.189	0.862	0.940	0.964	0.988
	Autoformer	0.852	0.924	0.987	1.034	1.267	1.417	1.687	1.797	1.020	1.077	1.173	1.215
	Informer	0.755	0.846	**0.829**	0.890	1.048	1.105	1.136	1.218	0.928	0.951	0.963	1.000
	iTransformer	0.762	0.888	0.936	0.971	1.186	1.487	1.629	1.706	0.987	1.104	1.153	1.184
	Transformer	0.712	0.814	0.913	0.925	0.961	1.186	1.310	1.392	0.889	0.986	1.034	1.069
	TimeFilter	**0.696**	0.807	0.885	0.935	1.008	1.218	1.388	1.462	0.910	0.999	1.064	1.096
	WPMixer	0.705	0.834	0.879	0.935	0.996	1.268	1.408	1.504	0.905	1.019	1.072	1.111
	FDMSNET	0.699	**0.797**	0.850	**0.878**	**0.892**	**1.050**	**1.132**	**1.181**	**0.856**	**0.927**	**0.961**	**0.985**
45	LSTM	0.843	0.908	0.906	0.912	0.999	1.222	1.158	1.176	0.906	1.001	0.972	0.983
	BiLSTM	0.844	0.889	0.901	0.908	1.008	1.109	1.149	1.218	0.910	0.953	0.968	1.000
	DLinear	0.704	0.806	0.852	0.887	0.896	1.064	1.138	1.193	0.858	0.934	**0.963**	0.990
	Autoformer	0.834	0.906	0.955	1.035	1.250	1.405	1.463	1.796	1.013	1.073	1.092	1.215
	Informer	0.799	0.811	0.847	0.880	1.137	1.159	1.139	**1.165**	0.967	0.974	0.964	**0.978**
	iTransformer	0.780	0.874	0.951	0.977	1.256	1.471	1.666	1.712	1.016	1.098	1.165	1.186
	Transformer	0.733	0.840	**0.835**	0.906	0.906	1.221	1.244	1.415	0.863	1.000	1.007	1.078
	TimeFilter	**0.690**	0.827	0.882	0.949	0.977	1.241	1.360	1.478	0.896	1.008	1.053	1.102
	WPMixer	0.723	0.833	0.902	0.941	1.016	1.281	1.426	1.498	0.913	1.025	1.078	1.109
	FDMSNET	0.702	**0.795**	0.842	**0.877**	**0.890**	**1.051**	**1.137**	1.186	**0.855**	**0.928**	**0.963**	0.987
2027	LSTM	0.907	0.884	0.905	0.916	1.218	1.108	1.179	1.186	1.000	0.953	0.980	0.987
	BiLSTM	0.844	0.908	0.898	0.927	1.000	1.222	1.151	1.188	0.906	1.001	0.969	0.988
	DLinear	0.704	0.802	**0.850**	0.886	0.898	1.074	1.141	1.187	0.859	0.938	0.965	0.988
	Autoformer	0.847	0.925	0.960	1.043	1.256	1.376	1.523	1.843	1.016	1.062	1.114	1.231
	Informer	0.769	0.888	0.858	0.901	1.016	1.187	1.137	1.211	0.914	0.986	0.963	0.998
	iTransformer	0.773	0.882	0.969	0.989	1.224	1.469	1.699	1.752	1.003	1.097	1.177	1.200
	Transformer	0.711	0.805	0.855	0.922	0.914	1.138	1.238	1.485	0.867	0.965	1.005	1.105
	TimeFilter	**0.688**	**0.795**	0.879	0.940	0.989	1.268	1.408	1.502	0.901	1.019	1.071	1.111
	WPMixer	0.709	0.814	0.915	0.962	1.000	1.245	1.437	1.521	0.906	1.010	1.082	1.118
	FDMSNET	0.699	**0.795**	0.857	**0.885**	**0.891**	**1.052**	**1.134**	**1.183**	**0.856**	**0.929**	**0.962**	**0.986**

In terms of the maximum improvement over a single model, FDMSNET achieves the largest reduction in MAE compared with Autoformer, reaching 14.39%. For MSE and RSE, the largest reductions are obtained compared with iTransformer, reaching 30.00% and 16.27%, respectively. This indicates that, in wind power scenarios characterized by strong randomness and multi-scale fluctuations, FDMSNET can more effectively extract key variation components through frequency decomposition and multi-scale modeling, thereby achieving more stable advantages in overall error control.

Fig 9 further presents the mean and standard deviation of different models on the wind power dataset across three random seeds. Each row corresponds to one prediction horizon, and each column corresponds to one evaluation metric. The bar height represents the mean error, while the error bar reflects the standard deviation across different random seeds. Therefore, a lower bar indicates better average forecasting accuracy, and a shorter error bar indicates stronger stability under different random initializations.

**Fig 9:**
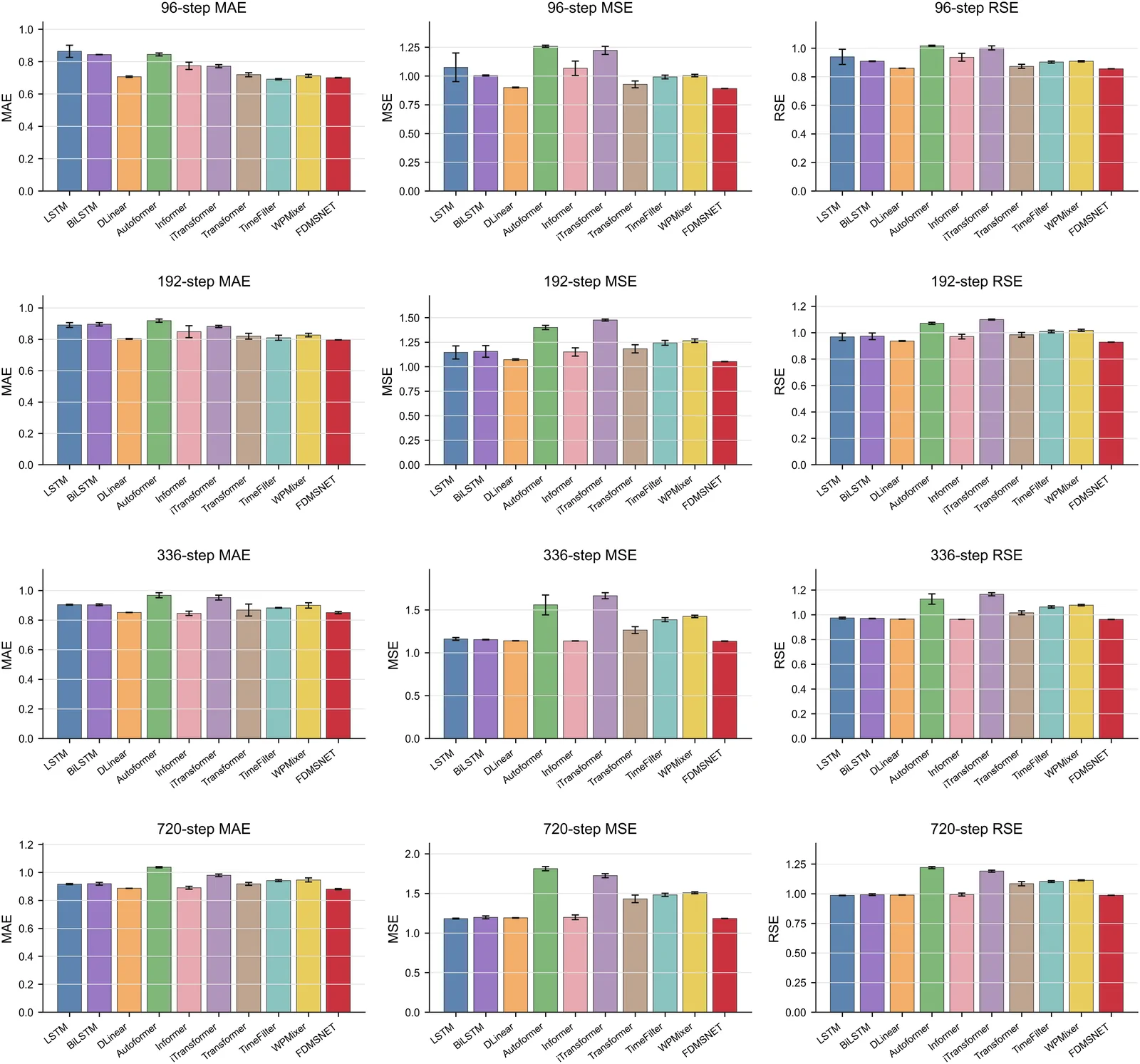
Mean and standard deviation of model errors on the wind power dataset. Rows correspond to prediction horizons and columns to MAE, MSE, and RSE. Bars show the mean across three random seeds, and error bars show the corresponding standard deviation.

Overall, FDMSNET maintains relatively low mean errors across most prediction horizons and evaluation metrics. In particular, for MSE and RSE, FDMSNET consistently achieves the lowest or near-lowest mean values under different horizons, indicating that it has strong advantages in controlling overall prediction error and relative error. Meanwhile, the error bars of FDMSNET are generally short, suggesting that its performance is less affected by random initialization and remains stable across the three seeds.

Although some baseline models achieve slightly lower MAE values under individual horizons, such as TimeFilter in short-term forecasting, their performance advantages are not consistent across all metrics and horizons. In contrast, FDMSNET shows more balanced behavior across MAE, MSE, and RSE. This indicates that the proposed frequency decomposition and multi-scale modeling strategy can improve both forecasting accuracy and robustness for wind power prediction.

### Solar Power Forecasting

As shown in Table 4, FDMSNET demonstrates strong overall forecasting accuracy and stability on the solar dataset across three random seeds and four prediction horizons. To provide a more comprehensive evaluation, the model performance is also compared from the perspective of the average errors of different model categories. The results show that, compared with recurrent neural network models, FDMSNET reduces the average MAE, MSE, and RSE by 49.59%, 45.49%, and 26.17%, respectively. Compared with the linear model, the average MAE, MSE, and RSE are reduced by 10.87%, 16.11%, and 8.40%, respectively. Compared with Transformer-based models, the corresponding reductions are 26.47%, 37.87%, and 20.30%. Compared with recent advanced time series forecasting models, the average MAE, MSE, and RSE are reduced by 6.81%, 13.36%, and 6.86%, respectively. These results indicate that FDMSNET not only clearly outperforms recurrent neural network models and Transformer-based models in solar power forecasting, but also maintains stable advantages over the linear model and recent advanced time series forecasting models.

**Table 4:** Performance comparison of different models on the solar power dataset across three random seeds.

Seed	Model	MAE				MSE				RSE			
		96	192	336	720	96	192	336	720	96	192	336	720
42	LSTM	0.567	0.592	0.591	0.594	0.422	0.470	0.448	0.455	0.688	0.730	0.717	0.722
	BiLSTM	0.571	0.594	0.592	0.588	0.422	0.453	0.449	0.448	0.689	0.716	0.718	0.717
	DLinear	0.301	0.330	0.353	0.350	0.258	0.290	0.315	0.303	0.538	0.573	0.601	0.589
	Autoformer	0.455	0.560	0.434	0.481	0.395	0.589	0.340	0.439	0.666	0.817	0.624	0.709
	Informer	0.325	0.402	0.406	0.381	0.290	0.374	0.389	0.356	0.571	0.651	0.668	0.639
	iTransformer	0.307	0.324	0.352	0.338	0.283	0.316	0.359	0.332	0.563	0.599	0.642	0.617
	Transformer	0.344	0.372	0.389	0.362	0.285	0.399	0.402	0.323	0.566	0.673	0.679	0.608
	TimeFilter	0.309	0.350	0.338	0.344	0.264	0.303	0.302	0.304	0.545	0.586	0.588	0.590
	WPMixer	**0.274**	0.299	**0.306**	**0.302**	0.235	0.274	0.273	0.264	0.514	0.557	0.560	0.551
	FDMSNET	0.277	**0.296**	0.309	0.305	**0.226**	**0.249**	**0.256**	**0.246**	**0.504**	**0.531**	**0.542**	**0.531**
45	LSTM	0.574	0.606	0.598	0.591	0.420	0.475	0.460	0.453	0.687	0.734	0.727	0.721
	BiLSTM	0.565	0.591	0.596	0.596	0.401	0.436	0.455	0.444	0.671	0.703	0.723	0.714
	DLinear	0.302	0.331	0.353	0.349	0.261	0.289	0.314	0.302	0.542	0.573	0.600	0.589
	Autoformer	0.542	0.578	0.391	0.673	0.506	0.602	0.308	0.617	0.754	0.826	0.595	0.841
	Informer	0.323	0.367	0.413	0.361	0.281	0.350	0.401	0.300	0.562	0.630	0.678	0.587
	iTransformer	0.300	0.320	0.359	0.335	0.276	0.312	0.365	0.327	0.557	0.595	0.647	0.612
	Transformer	0.321	0.356	0.372	0.353	0.297	0.351	0.383	0.311	0.577	0.630	0.663	0.598
	TimeFilter	0.311	0.329	0.364	0.337	0.266	0.289	0.315	0.302	0.546	0.572	0.601	0.589
	WPMixer	**0.273**	0.307	0.318	0.307	0.239	0.282	0.282	0.271	0.518	0.566	0.569	0.557
	FDMSNET	0.275	**0.298**	**0.309**	**0.306**	**0.225**	**0.249**	**0.256**	**0.246**	**0.503**	**0.531**	**0.542**	**0.532**
2027	LSTM	0.583	0.603	0.607	0.606	0.427	0.468	0.482	0.476	0.693	0.728	0.744	0.739
	BiLSTM	0.578	0.592	0.589	0.583	0.427	0.470	0.452	0.441	0.692	0.729	0.721	0.711
	DLinear	0.301	0.329	0.353	0.349	0.258	0.288	0.314	0.302	0.539	0.572	0.600	0.589
	Autoformer	0.561	0.673	0.410	0.868	0.567	0.778	0.341	1.005	0.798	0.939	0.626	1.073
	Informer	0.365	0.364	0.383	0.376	0.353	0.334	0.395	0.356	0.629	0.615	0.673	0.639
	iTransformer	0.299	0.323	0.346	0.344	0.279	0.315	0.350	0.339	0.560	0.597	0.634	0.623
	Transformer	0.346	0.360	0.398	0.386	0.351	0.361	0.449	0.438	0.628	0.639	0.718	0.709
	TimeFilter	0.316	0.364	0.356	0.351	0.275	0.314	0.324	0.317	0.556	0.596	0.610	0.603
	WPMixer	0.279	**0.297**	0.313	0.309	0.242	0.272	0.279	0.278	0.521	0.555	0.566	0.565
	FDMSNET	**0.276**	0.298	**0.310**	**0.307**	**0.226**	**0.249**	**0.257**	**0.246**	**0.504**	**0.531**	**0.543**	**0.531**

In terms of the maximum improvement over a single model, FDMSNET achieves the largest reduction in MAE compared with LSTM, reaching 49.86%. For MSE and RSE, the largest reductions are obtained compared with Autoformer, reaching 54.82% and 31.75%, respectively. This indicates that, in solar power forecasting scenarios, FDMSNET can more effectively capture the main variation patterns driven by solar irradiance and diurnal cycles, and reduce the overall errors across different prediction horizons through frequency decomposition and multi-scale modeling.

Fig 10 further presents the mean and standard deviation of different models on the solar dataset across three random seeds. Each row corresponds to one prediction horizon, and each column corresponds to one evaluation metric. The bar height represents the average error over the three random seeds, while the error bar denotes the standard deviation among different seeds. Therefore, a lower bar indicates a smaller average forecasting error, and a shorter error bar indicates that the model is less affected by random initialization and has more stable forecasting results.

**Fig 10:**
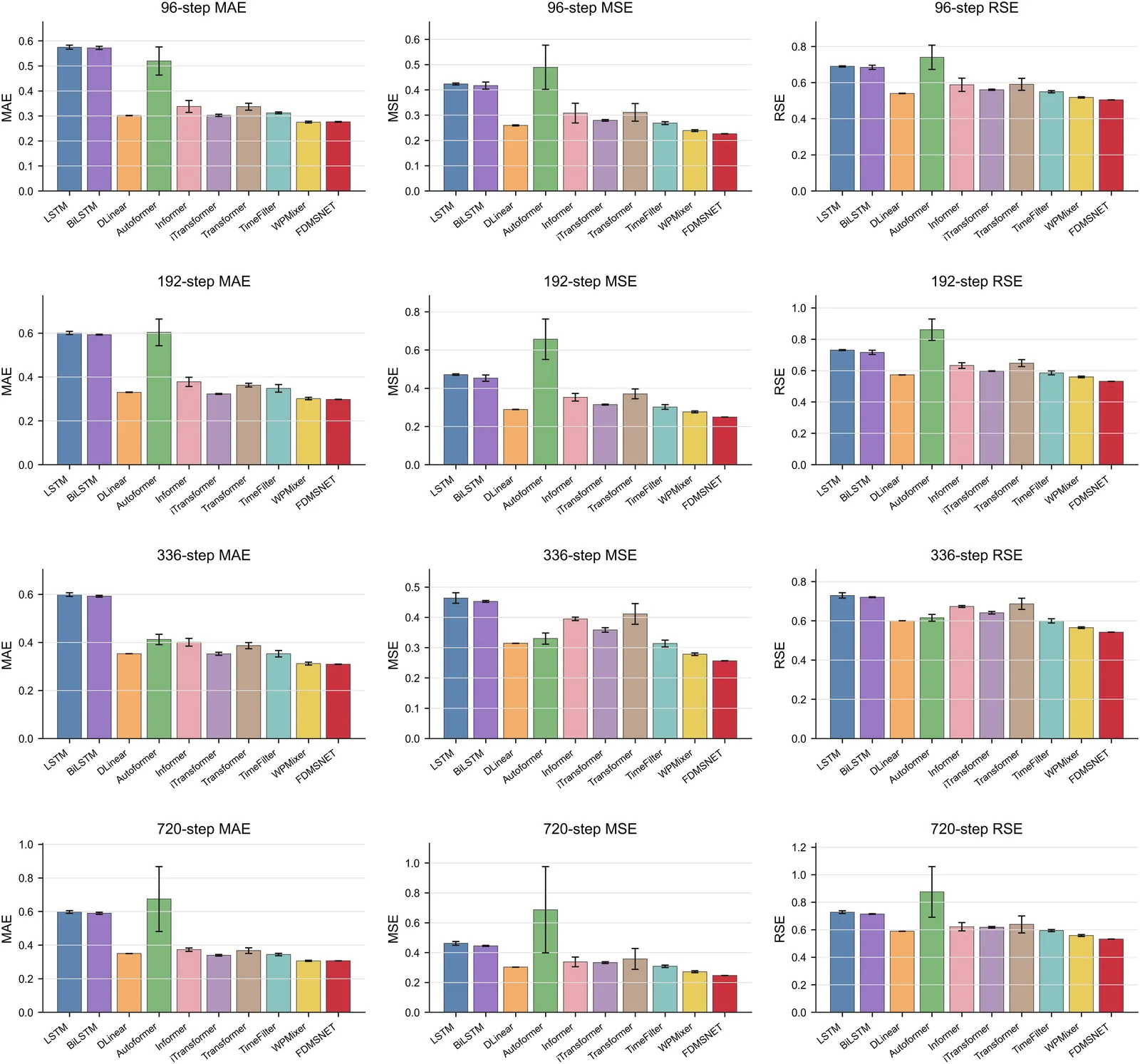
Mean and standard deviation of model errors on the solar power dataset. Rows correspond to prediction horizons and columns to MAE, MSE, and RSE. Bars show the mean across three random seeds, and error bars show the corresponding standard deviation.

Overall, FDMSNET shows low average errors and strong stability on the solar dataset. In particular, for MSE and RSE, FDMSNET achieves the lowest or near-lowest mean values across the four prediction horizons, indicating that the model can effectively control both overall error and relative error. Meanwhile, the error bars of FDMSNET are generally short, suggesting that its performance varies only slightly across the three random seeds and is insensitive to random initialization.

Compared with other models, some baselines perform better on individual MAE indicators; for example, WPMixer achieves lower MAE under some prediction horizons. However, this advantage is mainly limited to a single metric and is not consistently maintained in MSE and RSE. In contrast, FDMSNET shows more balanced performance across MAE, MSE, and RSE, and remains stable under different prediction horizons. This demonstrates that the proposed frequency decomposition and multi-scale modeling strategy can not only improve solar power forecasting accuracy, but also enhance robustness under different random initialization conditions.

In summary, the comparative experimental results show that FDMSNET achieves strong forecasting accuracy, stability, and generalization ability in both wind and solar power forecasting tasks. Compared with recurrent neural network models, the linear model, Transformer-based models, and recent advanced time series forecasting models, FDMSNET obtains lower average errors under most prediction horizons and evaluation metrics, while maintaining smaller performance fluctuations across multiple random seeds. Although some baseline models show local advantages under individual prediction horizons or specific metrics, FDMSNET demonstrates more balanced and stable overall performance when considering the comprehensive results across datasets, prediction horizons, and evaluation metrics. This indicates that the proposed frequency decomposition and multi-scale modeling strategy can effectively improve renewable energy power forecasting performance, and also provides reliable experimental evidence for subsequent average ranking and significance analyses.

### Ranking and significance analysis

To obtain more convincing model ranking results, this study introduces the Friedman overall comparison method based on the comparative experimental results, and further combines it with the Nemenyi post-hoc test to statistically analyze the performance differences among different models. Compared with evaluations based only on a single dataset, a single prediction horizon, or a single evaluation metric, average ranking and significance analysis can comprehensively assess model performance across datasets, prediction horizons, random seeds, and evaluation metrics, thereby reducing the influence of local optimal results on the overall conclusion.

### Average ranking analysis

In this study, both the wind and solar datasets are jointly included in the average ranking analysis. The experimental blocks are constructed from 2 datasets, 3 random seeds, 4 prediction horizons, and 3 evaluation metrics, resulting in a total of *N* = 72 ranking tasks. For each experimental block, all models are ranked according to the error metric values, where a smaller error corresponds to a better rank. The average rank of model *m* is defined as:


$$\begin{array}{c} {\overset{―}{R}}_{m}=\frac{1}{N}\sum_{b=1}^{N}r_{m,b}, \end{array}$$ (34)

where $r_{m,b}$ denotes the rank of model *m* in the *b*-th experimental block, and *N* denotes the total number of experimental blocks. A smaller average rank indicates better overall performance under the complete experimental setting.

To intuitively present the ranking performance of different models across wind and solar metrics, a cross-scenario average ranking radar chart is plotted. The radar chart contains six axes, corresponding to the MAE, MSE, and RSE rankings on the wind dataset, and the MAE, MSE, and RSE rankings on the solar dataset. Since a smaller rank indicates better performance, the radial coordinates in the figure range from 10 to 1 from the center outward; that is, a position closer to the outer edge indicates a better average rank and better model performance.

From the overall average ranking results, FDMSNET achieves an average rank of 1.24, ranking first among all models. DLinear, WPMixer, and TimeFilter obtain average ranks of 3.11, 4.29, and 4.96, respectively, showing relatively strong performance. Informer and Transformer obtain average ranks of 5.43 and 5.71, respectively, while Autoformer obtains an average rank of 8.92, indicating relatively weaker overall performance. These results show that FDMSNET achieves the best comprehensive ranking performance across both wind and solar scenarios.

As further shown in Fig 11, FDMSNET is located at or near the outermost position on all six radar axes, indicating that it maintains leading performance on wind MAE, MSE, and RSE, as well as solar MAE, MSE, and RSE. Specifically, the average ranks of FDMSNET on wind MAE, MSE, and RSE are 1.67, 1.17, and 1.17, respectively, while its average ranks on solar MAE, MSE, and RSE are 1.42, 1.00, and 1.00, respectively. In contrast, DLinear ranks well on wind-related metrics but declines on solar-related metrics, whereas WPMixer performs strongly on solar-related metrics but ranks relatively lower on wind-related metrics. This indicates that the advantage of FDMSNET does not rely on a single dataset or a single evaluation metric; instead, it maintains a more balanced and stable leading performance across wind and solar scenarios with different fluctuation characteristics.

**Fig 11:**
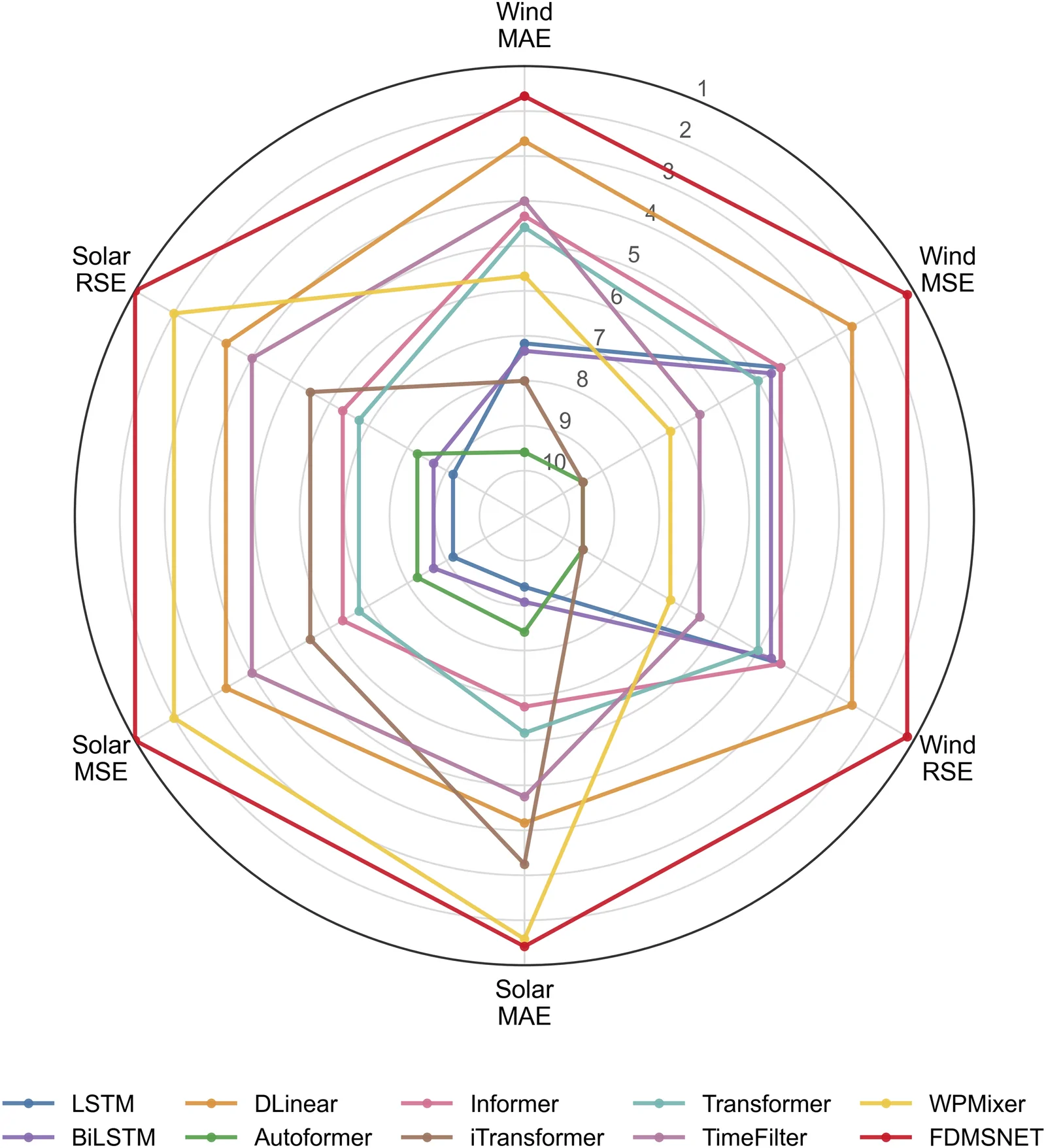
Cross-scenario average ranking radar chart. The six axes represent MAE, MSE, and RSE ranks for the wind and solar datasets. Ranks are reversed from 10 at the center to 1 at the outer edge, so positions nearer the edge indicate better performance.

### Significance analysis

After obtaining the average ranks, the Friedman test is further adopted to determine whether there are overall significant differences among different models. The Friedman test statistic is defined as:


$$\begin{array}{c} \chi_{F}^{2}=\frac{12N}{k(k+1)}\left[\sum_{m=1}^{k}{\overset{―}{R}}_{m}^{2}-\frac{k(k+1{)}^{2}}{4}\right], \end{array}$$ (35)

where *N* is the number of experimental blocks, *k* is the number of models, and ${\overset{―}{R}}_{m}$ is the average rank of model *m*. The null hypothesis of the Friedman test assumes that all models have the same forecasting performance, meaning that the differences in average ranks among models are not significant.

In this experiment, the Friedman statistic is 355.72, and the p-value is $3.76\times 10^{-71}$, which is far below the significance level of 0.05. Therefore, the null hypothesis is rejected, indicating that the overall performance differences among different models are statistically significant. Furthermore, the Nemenyi post-hoc test is used to compare the rank differences between FDMSNET and the other models. The critical difference is defined as:


$$\begin{array}{c} CD=q_{\alpha }\sqrt{\frac{k(k+1)}{6N}}. \end{array}$$ (36)

At the significance level of α=0.05, the critical difference in this experiment is 1.60. When the average-rank difference between two models is greater than this critical difference, the two models are considered to be significantly different.

As shown in Fig 12, the horizontal bar chart presents the average-rank differences between each baseline model and FDMSNET, while the dashed line denotes the Nemenyi critical difference of *CD* = 1.60. If a bar exceeds this dashed line, the average-rank difference between the corresponding model and FDMSNET reaches the significance level. The results show that the rank differences between DLinear, WPMixer, TimeFilter, Informer, Transformer, iTransformer, BiLSTM, LSTM, and Autoformer and FDMSNET are 1.88, 3.06, 3.72, 4.19, 4.47, 5.64, 5.94, 6.06, and 7.68, respectively, all exceeding the critical difference of 1.60. Therefore, FDMSNET has a statistically significant overall performance advantage over all baseline models.

**Fig 12:**
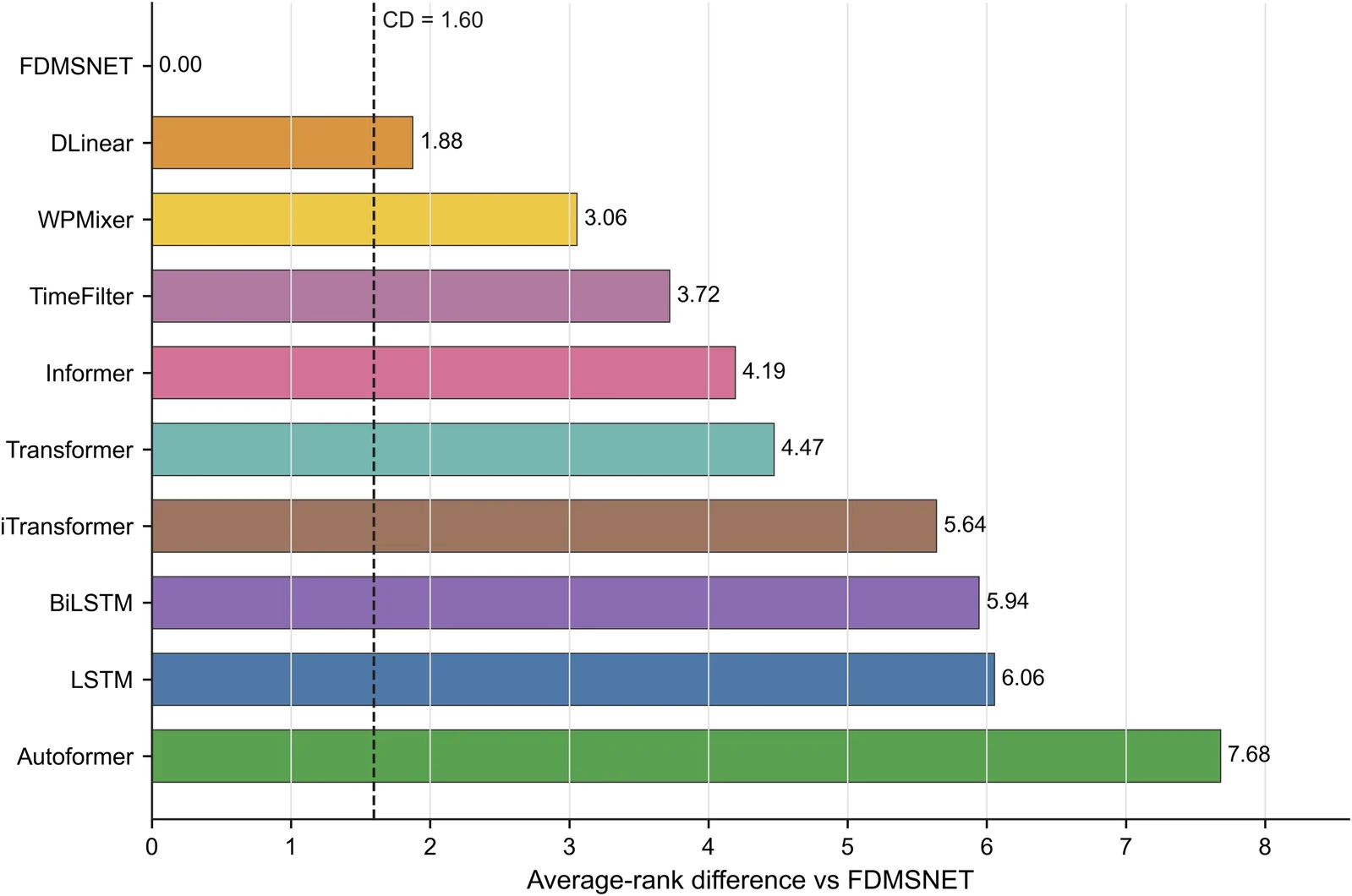
Nemenyi post-hoc comparison of FDMSNET with baseline models. Horizontal bars show the average-rank difference between each baseline and FDMSNET. The dashed line marks the Nemenyi critical difference (*CD* = 1.60); bars beyond this line indicate statistically significant differences at α=0.05.

In summary, the cross-scenario average ranking analysis and significance analysis further verify the comprehensive advantages of FDMSNET in renewable energy power forecasting tasks. FDMSNET maintains the best average rank across the wind and solar datasets, multiple prediction horizons, multiple random seeds, and multiple evaluation metrics, and shows significant differences compared with all baseline models. These results indicate that the proposed frequency decomposition and multi-scale modeling strategy not only improves forecasting accuracy under local experimental conditions, but also maintains stable and reliable performance advantages in cross-scenario comprehensive comparisons.

### Ablation experiment

Three ablation experiments were conducted to evaluate the contribution of each module in FDMSNET:

- **w/o_FD**: The frequency decomposition module is removed to evaluate the impact of smoothed data on forecasting accuracy.
- **w/o_ms**: The multi-scale module for the seasonal component is replaced with the original single-scale data to assess the effect of multi-scale information.
- **w/o_trend**: The prediction of the decomposed trend component is removed to examine its contribution to overall performance.

The ablation results are presented in Tables 5 and 6. A downward arrow (↓) denotes performance degradation relative to the full FDMSNET model, while values without arrows represent comparable performance.

**Table 5:** Ablation results on the wind dataset.

Model	pred_len	MAE	MSE	RSE
FDMSNET	96	0.697	0.891	0.855
w/o_FD	96	0.706↓	0.901↓	0.860↓
w/o_ms	96	0.699↓	0.891	0.855
w/o_trend	96	0.849↓	1.098↓	0.950↓
FDMSNET	192	0.799	1.055	0.930
w/o_FD	192	0.801↓	1.058↓	0.931↓
w/o_ms	192	0.799	1.057↓	0.931↓
w/o_trend	192	0.874↓	1.155↓	0.973↓
FDMSNET	336	0.844	1.135	0.962
w/o_FD	336	0.851↓	1.139↓	0.964↓
w/o_ms	336	0.846↓	1.136↓	0.963↓
w/o_trend	336	0.894↓	1.193↓	0.986↓
FDMSNET	720	0.887	1.182	0.986
w/o_FD	720	0.887	1.193↓	0.990↓
w/o_ms	720	0.887	1.185↓	0.986
w/o_trend	720	0.901↓	1.204↓	0.995↓

**Table 6:** Ablation results on the solar dataset.

Model	pred_len	MAE	MSE	RSE
FDMSNET	96	0.276	0.226	0.504
w/o_FD	96	0.307↓	0.259↓	0.540↓
w/o_ms	96	0.277↓	0.227↓	0.505↓
w/o_trend	96	0.761↓	0.890↓	1.000↓
FDMSNET	192	0.298	0.248	0.530
w/o_FD	192	0.334↓	0.293↓	0.576↓
w/o_ms	192	0.300↓	0.249↓	0.531↓
w/o_trend	192	0.758↓	0.884↓	1.001↓
FDMSNET	336	0.310	0.258	0.544
w/o_FD	336	0.355↓	0.314↓	0.600↓
w/o_ms	336	0.311↓	0.258	0.544
w/o_trend	336	0.754↓	0.873↓	1.001↓
FDMSNET	720	0.308	0.246	0.531
w/o_FD	720	0.349↓	0.302↓	0.589↓
w/o_ms	720	0.310↓	0.247↓	0.532↓
w/o_trend	720	0.755↓	0.874↓	1.001↓

Analysis of the ablation results indicates that removal of the trend prediction consistently leads to substantial performance degradation across all prediction horizons and datasets. Both MAE and MSE increase noticeably when the trend component is omitted, with the largest declines observed at longer horizons. This demonstrates that trend forecasting is a critical element in FDMSNET, providing essential information about the long-term temporal evolution of wind and solar power. Without this component, the model loses the ability to capture systematic variations over time, which significantly diminishes overall predictive accuracy.

The elimination of the frequency decomposition module generally results in moderate performance decreases in both wind and solar datasets. Metrics such as MAE, MSE, and RSE show slight but consistent increases relative to the full model. This indicates that frequency-domain smoothing contributes to more stable and accurate predictions by reducing high-frequency noise in the input data. Similarly, removing the multi-scale module for the seasonal component produces minor performance declines in most cases, suggesting that multi-scale seasonal processing enhances the model’s capability to capture variations at different temporal scales. These observations collectively confirm that both the frequency decomposition and multi-scale modules provide meaningful improvements to FDMSNET, even if the effect is less pronounced than that of trend prediction.

No ablation variant demonstrates any improvement over the complete FDMSNET model, confirming that the combination of trend prediction, frequency decomposition, and multi-scale seasonal processing constitutes the optimal module configuration. The observed performance declines upon removal of any individual module provide empirical evidence that each component plays a distinct and complementary role. Trend prediction ensures accurate long-term dynamics, frequency decomposition stabilizes high-frequency fluctuations, and multi-scale seasonal processing captures temporal patterns at multiple scales. Together, these modules form a synergistic design that maximizes forecasting performance across multiple horizons and datasets.

### Small-sample experiment

To further verify the forecasting capability of FDMSNET under limited training samples, this study conducts small-sample comparative experiments. The purpose of this experiment is to evaluate the performance changes of different time series forecasting models when the amount of available training data is significantly reduced, and to examine whether FDMSNET can still maintain good forecasting accuracy and stability under limited-sample conditions. In practical renewable energy stations, problems such as insufficient historical data, limited sample accumulation in newly built stations, or incomplete data collection often occur. Therefore, the small-sample experiment can further reflect the adaptability and robustness of the model in real-world applications.

In terms of experimental settings, 5%, 10%, 15%, and 20% of the original training set are selected as small-sample training data, while the validation set, test set, and forecasting task settings remain unchanged. All models involved in the comparison adopt the same core parameter settings as those in the main experiments to ensure comparability. The prediction horizons are still set to 96, 192, 336, and 720, and MAE, MSE, and RSE are used as evaluation metrics, where smaller values indicate better forecasting performance. By comparing results under different training sample ratios, the performance trends of models as the sample size gradually increases can be observed, and their sensitivity to training data size can be analyzed.

Tables 7 and 8 present the complete forecasting results on the wind and solar datasets under 5%, 10%, 15%, and 20% training sample ratios, respectively. The results show that, under small-sample conditions, different models exhibit clear performance differences across datasets, prediction horizons, and evaluation metrics. On the wind dataset, FDMSNET achieves relatively low errors under multiple training ratios and prediction horizons, and remains highly competitive compared with the average performance of all baseline models, indicating its good adaptability in sample-limited wind power forecasting scenarios. On the solar dataset, however, the absolute errors of FDMSNET are not always lower than those of DLinear, WPMixer, TimeFilter, or iTransformer, suggesting that some baseline models can also perform well in solar small-sample scenarios with clear diurnal patterns. Therefore, the small-sample experimental results more reasonably indicate that FDMSNET is not absolutely optimal under all local conditions, but it maintains good overall competitiveness and sample adaptability on both wind and solar datasets.

**Table 7:** Complete small-sample forecasting results on the wind dataset.

Training ratio	Model	MAE					MSE					RSE				
		96	192	336	720	Mean	96	192	336	720	Mean	96	192	336	720	Mean
5%	LSTM	0.925	0.986	1.047	1.064	1.005	1.620	1.804	1.975	1.986	1.846	0.957	1.009	1.053	1.060	1.020
	BiLSTM	0.922	0.991	1.043	**1.034**	0.997	1.618	**1.720**	1.980	**1.933**	**1.813**	0.956	**0.985**	1.054	**1.045**	**1.010**
	DLinear	0.889	**0.961**	**1.011**	1.044	**0.976**	**1.591**	1.797	**1.922**	1.993	1.826	**0.948**	1.007	**1.038**	1.061	1.014
	Autoformer	2.067	1.289	1.302	1.315	1.493	5.571	2.693	2.840	2.918	3.505	1.775	1.232	1.262	1.284	1.388
	Informer	1.911	3.288	2.874	2.082	2.539	5.688	14.940	11.496	6.754	9.719	1.793	2.902	2.540	1.954	2.297
	iTransformer	0.903	1.037	1.151	1.194	1.071	1.740	2.118	2.455	2.585	2.224	0.992	1.093	1.174	1.209	1.117
	Transformer	1.148	1.395	1.269	1.185	1.249	2.332	3.102	2.686	2.330	2.613	1.148	1.323	1.228	1.148	1.212
	TimeFilter	0.879	1.030	1.097	1.156	1.041	1.666	2.041	2.297	2.475	2.120	0.971	1.073	1.135	1.183	1.090
	WPMixer	**0.864**	1.020	1.113	1.181	1.044	1.617	2.076	2.351	2.569	2.153	0.956	1.082	1.149	1.205	1.098
	FDMSNET	0.899	0.975	1.012	1.035	0.980	1.683	1.920	2.020	2.128	1.938	0.975	1.040	1.065	1.097	1.044
10%	LSTM	0.928	0.999	1.032	1.040	1.000	1.388	1.577	**1.646**	**1.737**	**1.587**	0.915	0.974	**0.993**	**1.024**	**0.976**
	BiLSTM	0.904	0.981	1.037	1.037	0.990	1.367	**1.550**	1.681	1.755	1.588	0.908	**0.966**	1.003	1.029	**0.976**
	DLinear	**0.820**	0.924	0.961	**0.980**	0.921	**1.361**	1.617	1.722	1.804	1.626	**0.906**	0.986	1.015	1.043	0.988
	Autoformer	2.154	1.170	1.222	1.258	1.451	5.961	2.301	2.543	2.648	3.363	1.896	1.177	1.234	1.264	1.393
	Informer	2.023	2.278	1.831	1.315	1.862	6.712	7.420	5.225	2.980	5.584	2.012	2.113	1.769	1.341	1.809
	iTransformer	0.858	0.985	1.058	1.106	1.002	1.587	1.930	2.167	2.279	1.991	0.979	1.078	1.139	1.173	1.092
	Transformer	0.954	1.069	1.231	1.079	1.083	1.686	1.867	2.260	2.205	2.005	1.009	1.060	1.163	1.153	1.096
	TimeFilter	0.890	0.982	1.051	1.091	1.004	1.584	1.870	2.036	2.192	1.921	0.978	1.061	1.104	1.150	1.073
	WPMixer	0.840	0.998	1.076	1.127	1.010	1.531	1.930	2.193	2.291	1.986	0.961	1.078	1.146	1.176	1.090
	FDMSNET	0.822	**0.913**	**0.959**	0.987	**0.920**	1.371	1.600	1.713	1.851	1.634	0.910	0.981	1.013	1.057	0.990
15%	LSTM	0.889	0.958	0.987	0.951	0.946	1.157	1.330	1.409	1.287	1.296	0.949	1.016	1.043	1.001	1.002
	BiLSTM	0.871	0.960	0.936	0.954	0.930	1.112	1.328	1.292	1.297	1.257	0.930	1.015	0.999	1.005	0.987
	DLinear	**0.724**	0.811	0.853	**0.877**	**0.816**	**1.003**	**1.167**	**1.242**	**1.282**	**1.173**	**0.883**	**0.952**	**0.979**	**0.999**	**0.953**
	Autoformer	1.116	0.994	1.119	1.047	1.069	1.787	1.712	1.847	1.925	1.818	1.179	1.153	1.195	1.224	1.188
	Informer	1.639	1.441	1.389	1.388	1.464	4.173	3.375	3.190	3.316	3.514	1.802	1.619	1.570	1.606	1.649
	iTransformer	0.745	0.873	0.943	0.987	0.887	1.196	1.494	1.704	1.779	1.544	0.965	1.077	1.147	1.177	1.091
	Transformer	0.812	0.931	0.973	1.021	0.934	1.347	1.566	1.590	1.627	1.532	1.024	1.102	1.108	1.125	1.090
	TimeFilter	0.731	0.871	0.961	0.977	0.885	1.123	1.414	1.622	1.677	1.459	0.935	1.048	1.119	1.142	1.061
	WPMixer	0.747	0.863	0.947	0.980	0.884	1.170	1.480	1.668	1.773	1.523	0.954	1.072	1.135	1.175	1.084
	FDMSNET	0.726	**0.810**	**0.851**	**0.877**	**0.816**	1.013	1.174	1.244	1.287	1.180	0.888	0.955	0.980	1.001	0.956
20%	LSTM	0.874	0.913	0.954	0.962	0.926	1.080	1.176	1.245	1.254	1.189	0.924	0.963	0.988	0.996	0.968
	BiLSTM	0.862	0.921	0.938	0.955	0.919	1.055	1.179	1.216	1.251	1.175	0.913	0.964	0.977	0.994	0.962
	DLinear	**0.725**	0.815	0.864	0.895	0.825	**0.964**	**1.119**	**1.195**	1.239	**1.129**	**0.873**	**0.939**	**0.968**	0.990	**0.943**
	Autoformer	1.099	1.013	0.995	1.065	1.043	1.723	1.693	1.707	1.851	1.743	1.167	1.155	1.157	1.210	1.172
	Informer	1.605	1.400	1.312	1.238	1.389	4.331	3.428	2.912	2.648	3.330	1.851	1.644	1.512	1.447	1.613
	iTransformer	0.750	0.869	0.941	0.975	0.884	1.153	1.443	1.639	1.709	1.486	0.955	1.067	1.134	1.163	1.080
	Transformer	0.828	0.900	0.950	0.971	0.912	1.300	1.475	1.509	1.481	1.441	1.014	1.079	1.088	1.082	1.066
	TimeFilter	0.731	0.868	0.925	0.995	0.880	1.091	1.406	1.513	1.663	1.418	0.929	1.053	1.090	1.147	1.055
	WPMixer	0.742	0.866	0.948	0.973	0.882	1.151	1.436	1.632	1.702	1.480	0.954	1.064	1.132	1.160	1.078
	FDMSNET	0.726	**0.812**	**0.861**	**0.890**	**0.822**	0.976	1.125	1.198	**1.238**	1.134	0.879	0.942	0.969	**0.989**	0.945

**Table 8:** Complete small-sample forecasting results on the solar dataset.

Training ratio	Model	MAE					MSE					RSE				
		96	192	336	720	Mean	96	192	336	720	Mean	96	192	336	720	Mean
5%	LSTM	0.861	0.840	0.881	0.870	0.863	1.262	1.297	1.500	1.428	1.372	0.933	0.950	1.028	1.004	0.979
	BiLSTM	0.894	0.823	0.882	0.863	0.865	1.375	1.233	1.478	1.399	1.371	0.974	0.927	1.021	0.993	0.979
	DLinear	0.668	0.736	0.829	0.909	0.786	0.821	0.970	1.167	1.292	1.063	0.753	0.822	0.907	0.954	0.859
	Autoformer	0.974	1.032	1.018	1.007	1.007	1.543	1.792	1.684	1.556	1.644	1.032	1.117	1.090	1.047	1.072
	Informer	1.378	1.177	1.290	1.058	1.226	2.948	2.386	2.874	1.913	2.530	1.427	1.289	1.424	1.161	1.325
	iTransformer	**0.424**	**0.482**	0.573	0.652	0.533	**0.494**	**0.615**	0.829	0.900	**0.710**	**0.584**	**0.655**	0.765	0.797	**0.700**
	Transformer	1.158	1.352	0.981	1.090	1.145	2.634	3.191	1.781	1.991	2.399	1.349	1.491	1.121	1.185	1.286
	TimeFilter	0.445	0.489	**0.561**	**0.578**	**0.518**	0.515	0.632	**0.816**	**0.899**	0.716	0.596	0.663	**0.759**	**0.796**	0.704
	WPMixer	0.467	0.506	0.578	0.641	0.548	0.539	0.642	0.869	0.955	0.751	0.610	0.669	0.783	0.820	0.721
	FDMSNET	0.743	0.793	0.836	0.880	0.813	1.272	1.407	1.543	1.579	1.450	0.937	0.990	1.043	1.055	1.006
10%	LSTM	0.668	0.645	0.686	0.669	0.667	0.691	0.614	0.706	0.668	0.670	0.746	0.706	0.762	0.741	0.739
	BiLSTM	0.692	0.677	0.654	0.661	0.671	0.750	0.686	0.624	0.648	0.677	0.777	0.746	0.716	0.730	0.742
	DLinear	0.436	0.464	0.497	0.539	0.484	0.453	0.511	0.571	0.612	0.537	0.603	0.644	0.685	0.709	0.660
	Autoformer	1.090	0.964	0.724	0.740	0.879	1.827	1.470	0.900	1.037	1.308	1.212	1.092	0.860	0.923	1.022
	Informer	1.024	0.949	1.022	1.134	1.032	1.636	1.801	1.917	2.035	1.847	1.147	1.209	1.255	1.293	1.226
	iTransformer	**0.369**	**0.416**	0.451	**0.429**	**0.416**	**0.397**	**0.466**	**0.511**	**0.489**	**0.466**	**0.565**	**0.615**	**0.648**	**0.634**	**0.615**
	Transformer	0.764	0.749	0.792	0.840	0.786	1.021	1.499	1.501	1.434	1.364	0.906	1.103	1.110	1.085	1.051
	TimeFilter	0.382	**0.416**	**0.444**	0.440	0.421	0.418	0.480	0.524	0.523	0.486	0.580	0.624	0.656	0.655	0.629
	WPMixer	0.413	0.451	0.457	0.459	0.445	0.430	0.510	0.545	0.538	0.506	0.588	0.643	0.669	0.665	0.641
	FDMSNET	0.525	0.564	0.593	0.650	0.583	0.637	0.737	0.838	0.966	0.795	0.716	0.773	0.830	0.891	0.802
15%	LSTM	0.684	0.698	0.717	0.710	0.702	0.726	0.761	0.810	0.790	0.772	0.702	0.721	0.749	0.739	0.728
	BiLSTM	0.693	0.718	0.681	0.691	0.696	0.756	0.828	0.706	0.740	0.758	0.716	0.752	0.699	0.716	0.721
	DLinear	0.463	0.491	0.512	0.506	0.493	0.515	0.578	0.631	0.613	0.585	0.591	0.629	0.661	0.652	0.633
	Autoformer	1.101	0.946	0.779	0.741	0.892	1.928	1.462	1.122	1.079	1.397	1.143	1.000	0.881	0.864	0.972
	Informer	0.916	0.979	0.910	1.196	1.000	1.888	1.818	1.896	2.236	1.960	1.131	1.115	1.145	1.244	1.159
	iTransformer	**0.410**	**0.451**	**0.473**	**0.454**	**0.447**	**0.481**	**0.553**	**0.607**	**0.570**	**0.553**	**0.571**	**0.615**	**0.648**	**0.628**	**0.615**
	Transformer	0.872	0.725	0.769	0.782	0.787	1.298	1.211	1.126	1.100	1.184	0.938	0.910	0.883	0.873	0.901
	TimeFilter	0.427	0.460	0.489	0.472	0.462	0.515	0.569	0.632	0.603	0.580	0.591	0.624	0.662	0.646	0.630
	WPMixer	0.430	0.466	0.497	0.488	0.470	0.505	0.581	0.645	0.624	0.589	0.585	0.630	0.668	0.657	0.635
	FDMSNET	0.527	0.558	0.574	0.580	0.560	0.629	0.702	0.760	0.777	0.717	0.653	0.693	0.725	0.733	0.701
20%	LSTM	0.766	0.791	0.815	0.783	0.789	1.025	1.112	1.229	1.074	1.110	0.766	0.801	0.847	0.792	0.801
	BiLSTM	0.773	0.786	0.807	0.759	0.781	1.079	1.084	1.190	0.986	1.085	0.786	0.791	0.834	0.759	0.792
	DLinear	0.514	0.547	0.571	0.565	0.549	0.622	0.701	0.770	0.754	0.712	0.596	0.636	0.671	0.663	0.642
	Autoformer	1.028	0.849	0.848	0.785	0.877	1.559	1.192	1.341	1.230	1.331	0.944	0.829	0.885	0.848	0.877
	Informer	1.011	0.962	0.967	1.023	0.991	1.967	2.136	2.144	2.033	2.070	1.061	1.110	1.119	1.090	1.095
	iTransformer	**0.447**	**0.492**	**0.520**	**0.508**	**0.492**	**0.561**	**0.655**	**0.718**	**0.688**	**0.656**	**0.567**	**0.615**	**0.648**	**0.634**	**0.616**
	Transformer	0.742	0.748	0.722	0.605	0.704	1.405	1.595	1.422	0.874	1.324	0.896	0.959	0.911	0.715	0.870
	TimeFilter	0.471	0.511	0.535	0.515	0.508	0.604	0.683	0.751	0.718	0.689	0.588	0.628	0.662	0.647	0.631
	WPMixer	0.473	0.500	0.535	0.535	0.511	0.595	0.679	0.759	0.738	0.693	0.584	0.626	0.666	0.656	0.633
	FDMSNET	0.563	0.593	0.613	0.612	0.595	0.714	0.793	0.864	0.866	0.809	0.639	0.676	0.710	0.711	0.684

Fig 13 shows the performance changes of different models from the perspective of error reduction when the training sample ratio increases from 5% to 20%, which is used to measure the ability of each model to utilize additional training samples. The results show that the MAE, MSE, and RSE reduction rates of FDMSNET on the wind dataset are 16.1%, 41.5%, and 9.5%, respectively, among which MSE decreases most significantly. This indicates that FDMSNET improves its ability to control large prediction errors as the training sample size increases. On the solar dataset, the MAE, MSE, and RSE reduction rates of FDMSNET reach 26.8%, 44.2%, and 32.0%, respectively, showing a more obvious overall decreasing trend and indicating that the model can further improve forecasting performance with more training samples. Although some baseline models show higher reduction rates on certain metrics, this may be related to their larger initial errors under the 5% sample ratio. Overall, FDMSNET exhibits a relatively stable error reduction trend on both wind and solar datasets, suggesting that it has not only certain adaptability under low-sample conditions, but also good potential for utilizing additional samples.

**Fig 13:**
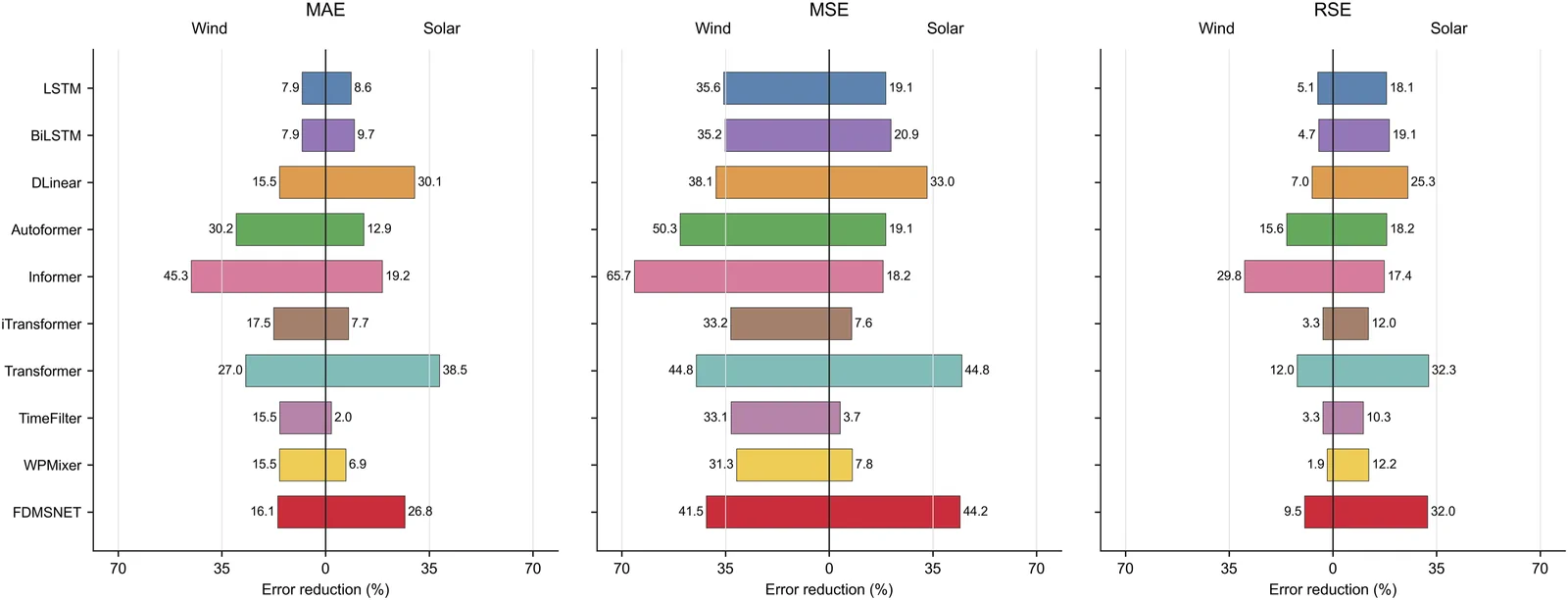
Error reduction from 5% to 20% training samples. The butterfly chart compares percentage reductions in MAE, MSE, and RSE for each model on the wind and solar datasets when the training proportion increases from 5% to 20%; larger positive values indicate greater error reduction.

## Conclusion

This paper proposes FDMSNet, a frequency-decomposed multi-scale hybrid forecasting framework, to address the non-stationarity, volatility, and multi-scale variation characteristics commonly observed in wind and photovoltaic power series. The proposed method first divides the original time series into non-stationary and relatively stationary components through a frequency decomposition module, thereby reducing the interference between different temporal patterns. Then, for the non-stationary component, a lightweight MLP branch is used to capture global nonlinear variation trends. For the stationary component, a multi-scale forecasting module is designed to model local fluctuations, periodic variations, and multi-scale dynamic features through multi-branch downsampling and cross-scale prediction fusion. Finally, the forecasting results of the two components are fused to obtain the final renewable energy power prediction.

Experimental results on real-world wind farm and photovoltaic power station datasets show that FDMSNet achieves good forecasting accuracy and stability under multiple prediction horizons. Compared with recurrent neural network models, linear decomposition models, Transformer-based models, and recently proposed advanced time-series forecasting models, FDMSNet achieves overall more competitive results in terms of MAE, MSE, and RSE. These results indicate that relying solely on recurrent structures, attention mechanisms, or fixed decomposition strategies is insufficient to fully characterize the complex non-stationary fluctuations in renewable energy power series, whereas the combination of frequency decomposition and multi-scale modeling can more effectively extract global variation trends, local periodic patterns, and multi-scale fluctuation features. In addition, average ranking analysis, significance testing, ablation experiments, and parameter sensitivity analysis further verify the effectiveness and robustness of the frequency decomposition module, the multi-scale modeling structure, and the key parameter settings.

Although FDMSNet achieves favorable forecasting performance in the overall experiments, it still has certain limitations in small-sample scenarios. When the number of training samples is limited, the advantage of the model is not obvious, and some results exhibit performance fluctuations. This may be because the frequency decomposition module and the multi-scale forecasting structure require sufficient data to learn stable frequency-domain components, local periodic patterns, and cross-scale variation rules. As the training sample size increases, the performance of FDMSNet gradually improves, indicating that the model is more suitable for renewable energy forecasting scenarios with relatively sufficient data. Future work will further investigate optimization strategies under small-sample conditions, such as data augmentation, cross-station pretraining, and integration with LLM-based models, to improve the stability and generalization capability of the model in small-sample scenarios. In addition, future research will consider incorporating richer meteorological information and extending the framework to multi-region, multi-station forecasting and probabilistic forecasting tasks.
