# Supplementary material for: FDMSNet: A frequency-decomposed multi-scale hybrid framework for renewable energy power forecasting
Source: PLoS One. 2026 Oct 13;21(10):e0356755. doi: 10.1371/journal.pone.0356755 (PMC13660931; doi:10.1371/journal.pone.0356755)
Supplement: Supplementary file 1 [file pone.0356755.s002.docx]

**PONE-D-26-16006**

**“FDMSNet: A Frequency-Decomposed Multi-Scale Hybrid Framework for Renewable Energy Power Forecasting”**

# **Reviewer #1 comments and our reply**

Reviewer #1:
The main contribution of the paper should be highlighted and emphasized. It would be great if the drawbacks and gaps in the literature were clear, and, particularly, how the proposed approach aims to address these gaps.
Comparative analysis needs to be incorporated.
Simulation Results should be enriched. More detailed scenarios should be added.
A separate section should be added to discuss the obtained results and main achievements.
The quality of all the figures is poor and should be improved.
References should be prepared according to the IEEE referencing style.
Try to reduce the similarity.
Include the following research articles in the Reference Section:
1. Gurugubelli, V., Ghosh, A. and Panda, A.K., 2022. A new virtual oscillator control for synchronization of single-phase parallel inverters in islanded microgrid. Energy Sources, Part A: Recovery, Utilization, and Environmental Effects, 44(4), pp.8842-8859.
2. Vikash, G., Ghosh, A. and Rudra, S., 2020, December. Integration of distributed generation to microgrid with virtual inertia. In 2020 IEEE 17th India council international conference (INDICON) (pp. 1-6). IEEE.
3. Gurugubelli, V., Ghosh, A. and Panda, A.K., 2023. Design and implementation of optimized andronov-hopf oscillator control method for parallel inverters in standalone microgrid. IEEE Transactions on Industry Applications, 59(6), pp.7013-7026.
4. Rana, N., Ghosh, A. and Banerjee, S., 2019. Design and implementation of an improved tri-state boost converter with optimal Type-III controller. International Journal of Power Electronics, 10(3), pp.236-265.
5. Ghosh, A., Sahu, A. and Barua, P., 2024. Particle Swarm Optimization-Based MPPT for PV Applications and Its Comparison With Incremental Conductance Method. In Optimization Techniques for Hybrid Power Systems: Renewable Energy, Electric Vehicles, and Smart Grid (pp. 308-324). IGI Global Scientific Publishing.

**Response to Reviewer 1:**

We sincerely thank the reviewer for the careful evaluation of our manuscript and for providing valuable and constructive comments. These suggestions have been very helpful in improving the clarity, completeness, and overall quality of the paper. **In response, we have revised the manuscript substantially, including strengthening the motivation and main contributions, expanding the comparative and supplementary experiments, adding a dedicated Discussion section, improving the quality and explanation of figures, checking the reference format and citations, reducing textual similarity, and incorporating relevant recommended literature.** We believe that these revisions have significantly improved the rigor, readability, and presentation of the manuscript. Detailed responses to each comment are provided below.

Detailed comments

**Comment 1:**The main contribution of the paper should be highlighted and emphasized. It would be great if the drawbacks and gaps in the literature were clear, and, particularly, how the proposed approach aims to address these gaps.
**Reply 1：**
Thank you very much for this valuable and constructive comment. We fully agree that clearly highlighting the main contribution and explicitly identifying the gaps in the existing literature are essential for strengthening the motivation of the study. The introductory sections have therefore been revised to separately examine the limitations of decomposition models—including traditional machine learning, RNNs, Transformers, DLinear/Autoformers—and multi-resolution models. The updated version further highlights that fixed-window decomposition struggles with temporal variations, multi-scale fluctuations, and irregular high-frequency disturbances characteristic of wind and solar power systems, while attention models are vulnerable to noise and data scale constraints. The contribution list explicitly emphasizes how FDMSNet addresses these challenges through frequency decomposition, component-specific modeling, and multi-scale integration.

**Comment 2:**Comparative analysis needs to be incorporated. **Reply 2:**
Thank you very much for this helpful suggestion. We agree that a more comprehensive comparative analysis is important for clearly demonstrating the effectiveness and robustness of the proposed method. The Comparative Experiments section has therefore been expanded. The revised version not only presents the result table, but also compares recent models—including RNNs, DLinear, Transformer architectures, and TimeFilter/WPMixer—by model category, with analysis covering MAE, MSE, RSE, varying prediction steps, performance across wind and solar datasets, and stability under different random seeds. **Details are provided in the “Comparative Experiments” chapter of the paper.**

**Comment 3:**Simulation Results should be enriched. More detailed scenarios should be added.
**Reply 3:**
The experimental scope has been significantly expanded. In addition to the original core experiments on wind power and photovoltaics, the revised version introduces three new sets of random seed results, visualizations of means and standard deviations, small-sample experiments, ablation experiments, Top-k parameter sensitivity analyses, as well as comprehensive rankings and significance analyses across datasets and metrics. These additions provide a more comprehensive evaluation of the proposed method. **Specific details are provided in the subsection following the “Discussion” section of the revised manuscript.**

**Comment 4:**A separate section should be added to discuss the obtained results and main achievements.
**Reply 4:**
Thank you very much for this valuable suggestion. We fully agree that a dedicated discussion section is important for clearly summarizing the obtained results, main findings, and achievements of the study. In the revised manuscript, **a separate Discussion section has been added**, which includes subsections such as Sensitivity Analysis, Comparative Experiments, Ranking and Significance Analysis, Ablation Experiment, and Small-Sample Experiment. These subsections provide a centralized discussion of the experimental results, key findings, model advantages, and limitations.

**Comment 5:**The quality of all the figures is poor and should be improved.
**Reply 5:**
Thank you very much for pointing this out. We agree that high-quality figures are essential for improving the readability and presentation of the experimental results. In the revised manuscript, all relevant figures have been regenerated and replaced, **including the data description figures, sensitivity analysis figures, mean/standard deviation bar charts, cross-scenario ranking radar charts, Friedman/Nemenyi post-hoc comparison figures, and small-sample experiment figures.** The figure captions, coordinate meanings, and analytical purposes have also been revised accordingly to make the graphical presentation clearer and more informative.

**Comment 6:**References should be prepared according to the IEEE referencing style.
**Reply 6:**
Thank you very much for this helpful reminder. We fully agree that the reference format should be consistent and carefully checked. Since this journal requires the PLOS ONE reference style, **the revised manuscript uses the PLOS-provided `plos2025.bst` bibliography style for formatting.** In addition, we have carefully checked the reference entries, numbering, and in-text citations to ensure consistency and to avoid the formatting inconsistencies present in the original version.

**Comment 7:**Try to reduce the similarity.
**Reply 7:**
Thank you very much for this important suggestion. We understand the reviewer’s concern and agree that reducing textual similarity is necessary to improve the originality and clarity of the manuscript. In the revised version, multiple expressions in the **Abstract, Introduction, Methodology, Experiment, Discussion,and Conclusion** have been rewritten to reduce template-like and repetitive wording. In addition, new experiments, extended discussions, and more specific methodological explanations have been added to increase the proportion of original content in the manuscript.

**Comment 8:**Include the following research articles in the Reference Section.
1. Gurugubelli, V., Ghosh, A. and Panda, A.K., 2022. A new virtual oscillator control for synchronization of single-phase parallel inverters in islanded microgrid. Energy Sources, Part A: Recovery, Utilization, and Environmental Effects, 44(4), pp.8842-8859.
2. Vikash, G., Ghosh, A. and Rudra, S., 2020, December. Integration of distributed generation to microgrid with virtual inertia. In 2020 IEEE 17th India council international conference (INDICON) (pp. 1-6). IEEE.
3. Gurugubelli, V., Ghosh, A. and Panda, A.K., 2023. Design and implementation of optimized andronov-hopf oscillator control method for parallel inverters in standalone microgrid. IEEE Transactions on Industry Applications, 59(6), pp.7013-7026.
4. Rana, N., Ghosh, A. and Banerjee, S., 2019. Design and implementation of an improved tri-state boost converter with optimal Type-III controller. International Journal of Power Electronics, 10(3), pp.236-265.
5. Ghosh, A., Sahu, A. and Barua, P., 2024. Particle Swarm Optimization-Based MPPT for PV Applications and Its Comparison With Incremental Conductance Method. In Optimization Techniques for Hybrid Power Systems: Renewable Energy, Electric Vehicles, and Smart Grid (pp. 308-324). IGI Global Scientific Publishing.
**Reply 8:**

Thank you very much for recommending these relevant research articles. We agree that incorporating appropriate studies can strengthen the background discussion and better connect the forecasting problem with practical power system operation and control issues.

**For example, in the introduction section of the main text:**

*“With the continuous acceleration of the transition toward low-carbon energy systems, renewable energy, especially wind and solar energy, is playing an increasingly important role in modern power systems ^[1]^. The large-scale integration of renewable energy contributes to reducing carbon emissions, optimizing the energy structure, and promoting the green transformation of power systems. However, wind and photovoltaic power generation are highly dependent on meteorological factors such as wind speed, solar irradiance, temperature, and cloud cover, and therefore exhibit significant intermittency, volatility, and uncertainty ^[2,3]^. These characteristics increase the complexity of power dispatch, reserve capacity allocation, and unit commitment, and may adversely affect the secure and stable operation of power systems. For example, in islanded microgrids and standalone power systems, rapid fluctuations in renewable energy output increase the regulation difficulty of power electronic devices such as inverters ^[4,5]^. In distributed power grids, unstable power injection may cause voltage fluctuations, frequency deviations, and stability issues ^[6]^. Meanwhile, the dynamic performance of power electronic converters can also be affected by rapid changes in renewable energy output ^[7]^. Therefore, developing accurate, stable, and highly generalizable wind and photovoltaic power forecasting models is of great significance for improving renewable energy accommodation capacity, power system operational efficiency, and microgrid control reliability ^[8]^.”*

# Reviewer #2 comments and our reply

**Reviewer #2:**
The manuscript titled “FDMSNet: A Frequency-Decomposed Multi-Scale Hybrid Framework for Renewable Energy Power Forecasting” proposes a hybrid forecasting model that combines frequency decomposition, an MLP-based trend branch, and a multi-scale seasonal branch for wind and solar power prediction. The topic is relevant to renewable-energy integration, power dispatch, and grid stability, and the paper fits the broad scientific and engineering scope of PLOS One. The authors compare FDMSNet with recurrent, linear, Transformer-based, and recent time-series baselines, reporting improvements in MAE, MSE, and RSE across wind and solar datasets. However, the manuscript requires major revision as several aspects of the mathematical formulation, dataset reporting, experimental design, statistical testing, and result interpretation should be further revised.

1. The introduction on pages 2–3 provides a broad survey of RNN, Transformer, DLinear, and hybrid forecasting methods, but the research gap remains too general. The manuscript states on page 3, lines 98–105, that existing models cannot sufficiently balance long-term trends and short-term fluctuations. Yet, it does not explain why decomposition-based models such as DLinear or Autoformer are inadequate for these specific renewable-energy datasets. This makes the proposed framework appear as an integration of known components rather than a clearly motivated methodological advance. The authors should add a focused comparison explaining the technical limitations of existing decomposition, attention, and multi-resolution models.

2. The multi-scale forecasting module on pages 6–7 contains redundant notation and a dimensional inconsistency. Equations 9 and 11 both define average pooling, while Equations 10 and 12 both define the same multi-scale sequence set, creating unnecessary repetition. More importantly, Equation 13 defines (W_0^{(i)} \in R^{H \times T}), even though pooled sequences at coarser scales should have reduced temporal lengths rather than the original length (T). The authors should revise the notation so that each scale has its own temporal dimension and explain how pooled outputs are aligned before averaging in Equation 14.

3. The evaluation metrics on page 9 are mathematically defined but operationally underspecified. Equations 22–24 define MAE, MSE, and RSE, while page 8 states that all features are standardised before modelling. The manuscript does not clarify whether the reported errors in Tables 2 and 3 are expressed in standardised units or converted back to physical units, such as megawatts. The final results should either be reported in physical units or supplemented with capacity-normalised metrics that power-system practitioners can interpret.

4. The baseline comparison protocol on pages 10–11 may not be fair. Table 1 assigns LSTM and Bi-LSTM a learning rate of 1E-5, while DLinear, Transformer variants, TimeFilter, WPMixer, and FDMSNet use 0.001. Without a documented validation search, this configuration could disadvantage recurrent baselines and inflate the apparent benefit of FDMSNet. The authors are encouraged to report the hyperparameter search space, batch size, number of epochs, early stopping rule, and number of training runs for each model.

5. The Top-k configuration seems inconsistent. Table 1 on page 11 lists FDMSNet with Top-k = 20, down-sampling layers = 3, and down-sampling window = 2. However, the sensitivity analysis on page 17, lines 462–468, concludes that the effective Top-k range is between 2 and 10 and that excessively large values provide minimal additional gains. The authors should explain why the main experiments use Top-k = 20 and confirm whether all reported results were generated with this setting.

6. Figures 4 and 5 do not match their textual interpretation. The captions describe “cumulative prediction error”, while the visuals on pages 12 and 13 show metric values across four discrete prediction horizons rather than cumulative errors or prediction trajectories. The surrounding text claims that FDMSNet closely follows the ground truth, but no ground-truth series or forecast overlay is shown. Please either relabel these figures as horizon-wise metric plots or add genuine time-series plots comparing predictions with observed power output.

7. The paired t-test procedure on pages 14–16 is statistically weak. The manuscript performs paired t-tests across only four horizons, as indicated by (H) in Equations 28–29, which is too small to represent the distribution of forecasting errors. Table 4 also indicates that the Bi-LSTM wind MSE comparison is significant despite a reported p-value of 0.053, which does not meet the stated (p < 0.05) threshold. The authors should redo the significance analysis using per-sample or repeated-run error distributions and apply multiple-comparison correction.

In summary, the manuscript addresses an important and timely problem in renewable-energy forecasting and is suitable for consideration by PLOS One because it presents an applied computational method for wind and solar power prediction. Nevertheless, it currently requires major revision before it can be considered methodologically sound and reproducible. Addressing the aforementioned issues would make the contribution more rigorous, interpretable, and useful for renewable energy forecasting research.

**Response to Reviewer 2:**

We sincerely thank the reviewer for the careful reading of our manuscript and for providing detailed, constructive, and highly valuable comments. These comments have helped us identify important issues in the motivation, mathematical notation, experimental settings, figure presentation, evaluation metrics, and statistical analysis. **In response, we have substantially revised the manuscript by clarifying the technical research gap, correcting the multi-scale forecasting notation, specifying the scale of the reported evaluation metrics, improving baseline and Top-k parameter settings, revising figure captions and interpretations, and replacing the original significance test with Friedman and Nemenyi analyses. We believe that these revisions have significantly improved the methodological clarity, experimental reliability, and overall presentation of the manuscript.**

Detailed comments

**Comment 1:**The introduction on pages 2–3 provides a broad survey of RNN, Transformer, DLinear, and hybrid forecasting methods, but the research gap remains too general. The manuscript states on page 3, lines 98–105, that existing models cannot sufficiently balance long-term trends and short-term fluctuations. Yet, it does not explain why decomposition-based models such as DLinear or Autoformer are inadequate for these specific renewable-energy datasets. This makes the proposed framework appear as an integration of known components rather than a clearly motivated methodological advance. The authors should add a focused comparison explaining the technical limitations of existing decomposition, attention, and multi-resolution models.
**Reply 1:**
Thank you very much for this insightful and constructive comment. We fully agree that the research gap in the original manuscript was not sufficiently focused, and that a clearer technical comparison is necessary to better justify the methodological motivation of the proposed framework. **In the revised manuscript, we have rewritten the method review and research-gap discussion in the Introduction. The revised version explicitly explains that the moving-average decomposition in DLinear is essentially fixed-window time-domain smoothing, which is difficult to adapt to time-varying frequency components; although Autoformer introduces a decomposition structure, it generally does not model different components according to their frequency characteristics; and Transformer-based models can be sensitive to noise, abrupt fluctuations, and data-scale limitations.** Based on these limitations, the revised manuscript further clarifies the rationale for the proposed technical route, namely frequency decomposition combined with component-specific forecasting and multi-scale fusion.

**Comment 2:**The multi-scale forecasting module on pages 6–7 contains redundant notation and a dimensional inconsistency. Equations 9 and 11 both define average pooling, while Equations 10 and 12 both define the same multi-scale sequence set, creating unnecessary repetition. More importantly, Equation 13 defines $(W_{0}^{(i)}\in R^{H\times T}),$ even though pooled sequences at coarser scales should have reduced temporal lengths rather than the original length $(T)$. The authors should revise the notation so that each scale has its own temporal dimension and explain how pooled outputs are aligned before averaging in Equation 14.
**Reply 2:**
Thank you very much for this careful and helpful comment. We agree that the original notation in the multi-scale forecasting module contained unnecessary repetition and did not clearly reflect the temporal-length changes caused by pooling. In the revised manuscript, we have rewritten the mathematical formulation of this module. Specifically, the temporal length of each scale is now defined as $T_{i}=T/w^{i}$, and the representation at the $i$-th scale is described as belonging to $B\times C\times T_{i}$. Accordingly, the scale-specific mapping matrix has been revised to $W_{\mathrm{sta}}^{(i)}\in{\mathbb{\mathbb{R}}}^{H\times T_{i}}$. Since each scale is directly mapped to the same prediction length $H$, the outputs of all scales have the unified shape $B\times H\times C$, allowing them to be directly fused along the prediction dimension.

**Comment 3:**The evaluation metrics on page 9 are mathematically defined but operationally underspecified. Equations 22–24 define MAE, MSE, and RSE, while page 8 states that all features are standardised before modelling. The manuscript does not clarify whether the reported errors in Tables 2 and 3 are expressed in standardised units or converted back to physical units, such as megawatts. The final results should either be reported in physical units or supplemented with capacity-normalised metrics that power-system practitioners can interpret.

Reply 3:
Thank you very much for this important and careful comment. We agree that the operational meaning of the reported metrics should be clearly specified, especially because standardization may affect the interpretation of forecasting errors. In the revised manuscript, we have added clarification in the Data Preprocessing and Evaluation Metrics sections. The revised version explicitly states that all features are standardized using the mean and standard deviation of the training set, and that the reported MAE, MSE, and RSE values are calculated and presented on the standardized scale. In addition, the dataset description has been supplemented with the installed capacities and power statistics of the wind and photovoltaic datasets to help readers better understand the data scale. **The corresponding clarification is provided in the “Data Description and Preprocessing” and “Evaluation Metrics” subsection.
For example, in the “Data Description and Preprocessing” and “Evaluation Metrics” section of the main text:**

***“The solar dataset is collected from Solar Station Site 3 with a nominal capacity of 30 MW, while the wind dataset is collected from Wind Farm Site 1 with a nominal capacity of 99 MW. Both datasets are recorded at 15-minute intervals and contain synchronized meteorological observations and renewable energy generation measurements. The solar dataset covers the period from January 1, 2019 to July 31, 2019, with 20,352 samples. It includes timestamp, total solar irradiance (TSI), direct normal irradiance (DNI), global horizontal irradiance (GHI), atmospheric pressure (Air_P), relative humidity (Air_H), and power output. The wind dataset covers the period from January 1, 2019 to December 31, 2020, with 70,176 samples. It includes timestamp, wind speed and wind direction at 10 m, 30 m, 50 m, and hub height, air temperature (Air_T), atmospheric pressure (Air_P), relative humidity (Air\_H), and power output. For the wind variables,***$\boldsymbol{WS\backslash\_\$x\$}$ ***and WD\_$x$ denote wind speed and wind direction measured at $x$ meters above the ground, respectively.The datasets are publicly available at https://github.com/Bob05757/Renewable-energy-generation-input-feature-variables-analysis/tree/main.”***

***“These metrics jointly evaluate the model’s accuracy, stability, and the relative error magnitude, providing a comprehensive assessment of forecasting performance across different horizons and datasets. All error metrics reported in this study are calculated and presented on the standardized scale.”***

**Comment 4:**The baseline comparison protocol on pages 10–11 may not be fair. Table 1 assigns LSTM and Bi-LSTM a learning rate of 1E-5, while DLinear, Transformer variants, TimeFilter, WPMixer, and FDMSNet use 0.001. Without a documented validation search, this configuration could disadvantage recurrent baselines and inflate the apparent benefit of FDMSNet. The authors are encouraged to report the hyperparameter search space, batch size, number of epochs, early stopping rule, and number of training runs for each model.
**Reply 4:**
Thank you very much for pointing out this important issue. We agree that consistent and fair hyperparameter settings are necessary to ensure the reliability of the comparative experiments. In the revised manuscript, the baseline parameter settings have been corrected. Specifically, the learning rates of LSTM and BiLSTM, which were set to$1\times10^{-5}$in the original version, have been uniformly adjusted to 0.001, making them consistent with the other baseline models and FDMSNet.

 **Comment 5:**The Top-k configuration seems inconsistent. Table 1 on page 11 lists FDMSNet with Top-k = 20, down-sampling layers = 3, and down-sampling window = 2. However, the sensitivity analysis on page 17, lines 462–468, concludes that the effective Top-k range is between 2 and 10 and that excessively large values provide minimal additional gains. The authors should explain why the main experiments use Top-k = 20 and confirm whether all reported results were generated with this setting.
**Reply 5:**
Thank you very much for this helpful suggestion. We agree that the main experimental settings should be consistent with the findings of the sensitivity analysis to ensure a fair and well-justified evaluation. In the revised manuscript, the main experimental parameters of FDMSNet have been corrected. Specifically, FDMSNet no longer uses a uniform Top-$k=20$ setting for all datasets; instead,Top-$k$ is set separately according to the dataset characteristics: $Top-k=8$for the wind power dataset and Top-$k$=1 for the photovoltaic dataset. The Sensitivity Analysis subsection in the Discussion explains that the wind power results become stable when Top-$k$is around 8, while the photovoltaic dataset performs better at Top-$k$=1. Therefore, this configuration is adopted in the subsequent comparative experiments.

**Comment 6:**Figures 4 and 5 do not match their textual interpretation. The captions describe “cumulative prediction error”, while the visuals on pages 12 and 13 show metric values across four discrete prediction horizons rather than cumulative errors or prediction trajectories. The surrounding text claims that FDMSNet closely follows the ground truth, but no ground-truth series or forecast overlay is shown. Please either relabel these figures as horizon-wise metric plots or add genuine time-series plots comparing predictions with observed power output.
**Reply 6:**
Thank you very much for pointing out this inconsistency. We agree that the figure captions and textual interpretation should accurately match the information shown in the plots. In the revised manuscript, the inaccurate descriptions such as “cumulative prediction error” and “closely follows the ground truth” have been removed. For the wind power and photovoltaic datasets, the revised **figure 9 and figure 10** captions now clearly state that the bar height represents the mean error over multiple random seeds, while the error bars represent the standard deviation. These revisions ensure that the figure titles, coordinate meanings, and textual explanations are consistent with the actual visual content.

**Comment 7:**The paired t-test procedure on pages 14–16 is statistically weak. The manuscript performs paired t-tests across only four horizons, as indicated by (H) in Equations 28–29, which is too small to represent the distribution of forecasting errors. Table 4 also indicates that the Bi-LSTM wind MSE comparison is significant despite a reported p-value of 0.053, which does not meet the stated (p < 0.05) threshold. The authors should redo the significance analysis using per-sample or repeated-run error distributions and apply multiple-comparison correction.
**Reply 7:**
Thank you very much for this rigorous and important comment. We agree that the original paired t-test analysis was statistically insufficient, especially because it was based on only four prediction horizons and contained an inconsistency in the significance annotation. **In the revised manuscript, the original paired t-test analysis has been replaced with a Friedman overall comparison followed by the Nemenyi post-hoc test.** The experimental blocks are constructed from 2 datasets, 3 random seeds, 4 prediction horizons, and 3 evaluation metrics, resulting in N=72 ranking tasks. The revised manuscript reports a Friedman statistic of 355.72 with $p=3.76\times10^{-71}$, and a Nemenyi critical difference of 1.60. This revised analysis avoids the small-sample limitation of the original horizon-based t-tests and corrects the previous significance-labeling issue. The details are provided in the **Ranking and Significance Analysis** subsection.

# Reviewer #3 comments and our reply

**Reviewer 3#:**
This manuscript addresses a relevant renewable-energy forecasting problem and the proposed decomposition-plus-multi-scale idea is reasonable. The paper has potential, but the current version still needs substantial technical revision in the model formulation, statistical validation, figure interpretation, and experimental fairness before the conclusions can be considered fully reliable.

1) The mathematical interpretation of the decomposition is not sufficiently convincing. The manuscript defines the trend component as the inverse transform of the top-k largest-amplitude frequency components, but in renewable-energy series, high-amplitude components are not necessarily “trend” in the usual time-series sense; they may also include dominant seasonal cycles. The paper should justify this choice more rigorously and compare it against a more standard low-frequency trend extraction strategy.

2) There is a dimensional inconsistency in the multi-scale module. After repeated average pooling, the temporal length of each scale branch changes, but Eq. (13) still defines the mapping matrix for every branch as W^(i) in R^(H×T). This should be corrected carefully, because the current formulation does not match the pooled sequence lengths.

3) The baseline comparison is not yet fully fair. In Table 1, LSTM and BiLSTM are trained with a learning rate of 1e-5, while most other baselines and the proposed model use 1e-3, and no hyperparameter tuning protocol is described. The reported superiority of FDMSNet is therefore difficult to interpret unless the baselines are tuned under comparably careful settings.

4) The literature positioning can be improved by discussing more recent work on data-efficient renewable-energy forecasting. In particular, the discussion would be strengthened by engaging with 10.1016/j.apenergy.2026.127625, especially in relation to forecasting under limited data, trend extraction, and modern architecture design for wind-power prediction.

5) The statistical testing is weak in its current form. The paired t-test is conducted across only four prediction horizons, which is a very small sample for a significance claim, and the test is applied to horizon-level summary errors rather than to rolling forecast errors. In addition, Table 4 appears to contain at least one incorrect significance mark, for example p = 0.053 is labeled with “*”. This entire significance analysis should be rechecked and ideally replaced or supplemented with a more appropriate forecasting comparison test.

6) The figures need clearer technical presentation. In the wind and solar result sections, the text says that Figs. 4 and 5 show prediction trajectories following the ground truth, but the captions describe them as cumulative prediction error plots. This inconsistency makes the visual evidence difficult to interpret. The authors should revise these figures and captions so that the plotted quantity, axes, units, and analytical purpose are unambiguous.

7) The result interpretation is too strong relative to the numerical margins in some tables. For example, in Table 2 the proposed model is only marginally better or tied with strong baselines in some horizons, yet the text repeatedly uses broad claims such as “consistently outperforms” and “superior generalization capability.” These statements should be moderated and discussed more carefully.

8) The average-rank analysis is not yet rigorous enough to support a strong ranking claim. The paper averages ranks across only four horizons and three metrics, and then visualizes them with a radar chart. This is descriptive but not a robust statistical ranking framework. The authors should consider a Friedman-type overall comparison with appropriate post-hoc testing if they want to make a stronger ranking claim.

**Response to Reviewer 2:**

Detailed comments

**Comment 1:** The mathematical interpretation of the decomposition is not sufficiently convincing. The manuscript defines the trend component as the inverse transform of the top-k largest-amplitude frequency components, but in renewable-energy series, high-amplitude components are not necessarily “trend” in the usual time-series sense; they may also include dominant seasonal cycles. The paper should justify this choice more rigorously and compare it against a more standard low-frequency trend extraction strategy.
**Reply 1:**
Thank you very much for this insightful and important comment. We agree that the top-k largest-amplitude frequency components should not be simply interpreted as the conventional “trend” component, since in renewable-energy time series they may also include dominant seasonal or periodic patterns. In the revised manuscript, we have therefore corrected the terminology and interpretation. **Specifically, these components are now described as dominant non-stationary information or the non-stationary component,** representing the dominant frequency components that contribute strongly to global variations, rather than a traditional low-frequency trend. The residual part is correspondingly interpreted as relatively stable local fluctuations and periodic information.

The reference links are as follows：

[Frequency adaptive normalization for non-stationary time series forecasting](https://proceedings.neurips.cc/paper_files/paper/2024/hash/37c6d0bc4d2917dcbea693b18504bd87-Abstract-Conference.html)

[Frequency-adaptive decomposition with KAN for medium-to-long-term wind power forecasting](https://www.sciencedirect.com/science/article/pii/S1568494626002127)

Following the terminology and motivation used in related frequency-adaptive studies, we have further clarified in the Introduction and Conclusion that fixed low-frequency extraction or fixed-window smoothing strategies may be insufficient for renewable-energy series with time-varying periodicity and multi-scale disturbances. Therefore, the proposed method adopts a data-driven dominant-frequency decomposition strategy to improve adaptability to such non-stationary characteristics.

**Comment 2:** There is a dimensional inconsistency in the multi-scale module. After repeated average pooling, the temporal length of each scale branch changes, but Eq. (13) still defines the mapping matrix for every branch as W^(i) in R^(H×T). This should be corrected carefully, because the current formulation does not match the pooled sequence lengths.
**Reply 2:**
Thank you very much for pointing out this dimensional inconsistency. We agree that the original formulation did not correctly reflect the change in temporal length after repeated average pooling, which could cause confusion in the mathematical description of the multi-scale module. In the revised manuscript, we have corrected the corresponding equations. Specifically, the temporal length of each scale is now denoted as $T_{i}$, and the mapping matrix for the $i$-th scale has been revised to $W_{\mathrm{sta}}^{(i)}\in{\mathbb{\mathbb{R}}}^{H\times T_{i}}$. We have also clarified that each scale is independently projected to the same prediction length $H$, so the outputs from different scales can be subsequently averaged or fused.

**Comment 3:** The baseline comparison is not yet fully fair. In Table 1, LSTM and BiLSTM are trained with a learning rate of 1e-5, while most other baselines and the proposed model use 1e-3, and no hyperparameter tuning protocol is described. The reported superiority of FDMSNet is therefore difficult to interpret unless the baselines are tuned under comparably careful settings.
**Reply 3:**
Thank you very much for this important and constructive comment. We agree that fair and consistent baseline settings are essential for making the comparative results reliable and interpretable. In the revised manuscript, the parameter settings in Table 1 have been corrected by changing the learning rates of LSTM and BiLSTM to 0.001, making them consistent with the other baseline models and the proposed FDMSNet. In addition, we have supplemented repeated experiments with multiple random seeds, so that the comparison results do not depend on a single training run and can better reflect the stability of each model.

 **Comment 4:** The literature positioning can be improved by discussing more recent work on data-efficient renewable-energy forecasting. In particular, the discussion would be strengthened by engaging with 10.1016/j.apenergy.2026.127625, especially in relation to forecasting under limited data, trend extraction, and modern architecture design for wind-power prediction.
Reply 4:
Thank you very much for this valuable suggestion. We agree that discussing recent work on data-efficient renewable-energy forecasting is important for better positioning the proposed method, especially under limited-data conditions. In the revised manuscript, we have supplemented the literature discussion by referring to recent related research, including the study suggested by the reviewer, and have further addressed the issue of data efficiency through additional experiments. Specifically, a new **Small-Sample Experiment** subsection has been added to compare model performance under 5%, 10%, 15%, and 20% training-sample ratios. In the Conclusion, we also acknowledge that FDMSNet does not always show an absolute advantage under extremely limited sample conditions, and we identify data augmentation and cross-site pretraining as promising directions for future work.

**Comment 5:** The statistical testing is weak in its current form. The paired t-test is conducted across only four prediction horizons, which is a very small sample for a significance claim, and the test is applied to horizon-level summary errors rather than to rolling forecast errors. In addition, Table 4 appears to contain at least one incorrect significance mark, for example p = 0.053 is labeled with “*”. This entire significance analysis should be rechecked and ideally replaced or supplemented with a more appropriate forecasting comparison test.
**Reply 5:**
Thank you very much for this careful and important comment. We agree that the original paired t-test was not sufficiently robust because it was based on only four prediction horizons and horizon-level summary errors, and we also appreciate the reviewer for pointing out the incorrect significance mark. In the revised manuscript, the original paired t-test results and the erroneous significance annotations have been removed. **We have replaced them with a Friedman overall comparison and a Nemenyi post-hoc test.** The new analysis is based on 72 experimental blocks covering datasets, random seeds, prediction horizons, and evaluation metrics, which provides a more robust assessment of the overall ranking differences among the compared models.

**Comment 6:** The figures need clearer technical presentation. In the wind and solar result sections, the text says that Figs. 4 and 5 show prediction trajectories following the ground truth, but the captions describe them as cumulative prediction error plots. This inconsistency makes the visual evidence difficult to interpret. The authors should revise these figures and captions so that the plotted quantity, axes, units, and analytical purpose are unambiguous.
**Reply 6:**
Thank you very much for pointing out this issue. We agree that the original figures could be misleading if discrete metric values were interpreted as prediction trajectories or cumulative errors. In the revised manuscript, the result figures have been reorganized. **The original cumulative-error-style figures have been replaced with mean/standard deviation plots organized by prediction horizon and evaluation metric, now presented as Figures 9 and 10. The revised text also clearly explains that the bar height represents the mean error and the error bars represent the standard deviation, thereby avoiding confusion between horizon-wise metric plots, cumulative errors, and prediction trajectories.**

**Comment 7:**The result interpretation is too strong relative to the numerical margins in some tables. For example, in Table 2 the proposed model is only marginally better or tied with strong baselines in some horizons, yet the text repeatedly uses broad claims such as “consistently outperforms” and “superior generalization capability.” These statements should be moderated and discussed more carefully.
**Reply 7:**
Thank you very much for this thoughtful and helpful comment. We agree that the result interpretation should be consistent with the actual numerical margins and should avoid overly broad claims. In the revised manuscript, we have moderated the relevant statements in the wind power and photovoltaic result analyses. Expressions such as “consistently outperforms” and “superior generalization capability” have been replaced or qualified with more cautious wording, including “lowest or near-lowest,” “more balanced performance,” and “some baselines perform better on individual MAE indicators.” Meanwhile, we have calculated and reported the specific improvement percentages where appropriate, so that the performance differences are described more quantitatively. We also explicitly acknowledge that some baseline models show local advantages under particular prediction horizons or evaluation metrics.
**For example, the following paragraph in the paper:**
*“As shown in Table 3, FDMSNET demonstrates strong overall forecasting capability on the wind power dataset across three random seeds and four prediction horizons. To provide a more comprehensive evaluation, the model performance is first compared from the perspective of the average errors of different model categories. The results show that, compared with recurrent neural network models, FDMSNET reduces the average MAE, MSE, and RSE by 9.59%, 6.09%, and 3.17%, respectively. Compared with the linear model, the average MAE, MSE, and RSE are reduced by 0.68%, 0.95%, and 0.48%, respectively. Compared with Transformer-based models, the corresponding reductions are 8.06%, 20.63%, and 10.66%. Compared with recent advanced time series forecasting models, the average MAE, MSE, and RSE are reduced by 3.84%, 17.29%, and 8.90%, respectively. These results indicate that FDMSNET not only achieves clear advantages over complex Transformer-based models, but also remains highly competitive against recurrent neural network models, the linear model, and recent advanced forecasting models.”*

**Comment 7:**The average-rank analysis is not yet rigorous enough to support a strong ranking claim. The paper averages ranks across only four horizons and three metrics, and then visualizes them with a radar chart. This is descriptive but not a robust statistical ranking framework. The authors should consider a Friedman-type overall comparison with appropriate post-hoc testing if they want to make a stronger ranking claim.

**Reply 8:**
Thank you very much for this valuable suggestion. We agree that the original average-rank analysis was mainly descriptive and was not sufficient to support a strong statistical ranking claim. **In the revised manuscript, we have adopted the reviewer’s suggestion and added a Friedman overall test together with the Nemenyi post-hoc test in the Ranking and Significance Analysis subsection.** The revised analysis reports the average ranks, the critical difference, and the rank differences relative to FDMSNet, thereby replacing the previous reliance on descriptive ranking plots based only on four prediction horizons and three metrics. This provides a more rigorous statistical basis for comparing the overall performance of the models.
